# Influence of extracellular matrix scaffolds on histological outcomes of regenerative endodontics in experimental animal models: a systematic review

**DOI:** 10.1186/s12903-024-04266-x

**Published:** 2024-04-30

**Authors:** Hisham Elnawam, Amr Abdallah, Samir Nouh, Nesma Mohamed Khalil, Rania Elbackly

**Affiliations:** 1 Endodontics, Conservative Dentistry Department, Faculty of Dentistry, Alexandria University, Champollion Street, Azarita, Alexandria Egypt; 2 Tissue Engineering Laboratories, Faculty of Dentistry, Alexandria University, Alexandria, Egypt; 3 Surgery Department, Faculty of Veterinary Medicine, Alexandria University, Alexandria, Egypt; 4 Oral Biology Department, Faculty of Dentistry, Alexandria University, Alexandria, Egypt

**Keywords:** Extracellular matrix, Biological scaffolds, Decellularization, Regenerative endodontics, Pulp regeneration, Experimental animals, Histology

## Abstract

**Background:**

Decellularized extracellular matrix (dECM) from several tissue sources has been proposed as a promising alternative to conventional scaffolds used in regenerative endodontic procedures (REPs). This systematic review aimed to evaluate the histological outcomes of studies utilizing dECM-derived scaffolds for REPs and to analyse the contributing factors that might influence the nature of regenerated tissues.

**Methods:**

The PRISMA 2020 guidelines were used. A search of articles published until April 2024 was conducted in Google Scholar, Scopus, PubMed and Web of Science databases. Additional records were manually searched in major endodontic journals. Original articles including histological results of dECM in REPs and in-vivo studies were included while reviews, in-vitro studies and clinical trials were excluded. The quality assessment of the included studies was analysed using the ARRIVE guidelines. Risk of Bias assessment was done using the (SYRCLE) risk of bias tool.

**Results:**

Out of the 387 studies obtained, 17 studies were included for analysis. In most studies, when used as scaffolds with or without exogenous cells, dECM showed the potential to enhance angiogenesis, dentinogenesis and to regenerate pulp-like and dentin-like tissues. However, the included studies showed heterogeneity of decellularization methods, animal models, scaffold source, form and delivery, as well as high risk of bias and average quality of evidence.

**Discussion:**

Decellularized ECM-derived scaffolds could offer a potential off-the-shelf scaffold for dentin-pulp regeneration in REPs. However, due to the methodological heterogeneity and the average quality of the studies included in this review, the overall effectiveness of decellularized ECM-derived scaffolds is still unclear. More standardized preclinical research is needed as well as well-constructed clinical trials to prove the efficacy of these scaffolds for clinical translation.

**Other:**

The protocol was registered in PROSPERO database #CRD42023433026. This review was funded by the Science, Technology and Innovation Funding Authority (STDF) under grant number (44426).

**Supplementary Information:**

The online version contains supplementary material available at 10.1186/s12903-024-04266-x.

## Background

The current research focus in the field of regenerative endodontics is to reach more "predictable" outcomes regarding the success rates of this treatment modality as well as the nature of the regenerated tissues which should ideally mimic the native dentin-pulp complex [1]. Regenerative Endodontic Procedures (REPs) require three critical elements including elimination of residual bacteria and bacterial antigens, a scaffold to support and promote stem cell adhesion and proliferation, and coronal seal using a biocompatible material [2, 3].

These procedures generally have two main approaches, the cell-based approach which involves the transplantation of stem cells from an exogenous source, and the cell homing approach which aims to harness the body's own response acting on endogenous residing progenitor cells and factors bypassing the need for exogenous delivery [4, 5]. While cell-based strategies have been advocated and have demonstrated appealing results, the true value of the transplanted cells has been debatable, and their presence does not always result in predictable tissue regeneration [6]. An area of considerable interest in REPs is the scaffold type and the interplay between scaffolds and mesenchymal stem cells [7]. Synthetic scaffolds have many advantageous biological properties such as biocompatibility, controlled biodegradability and mild inflammatory response [8]. Additionally, their mechanical properties, viscosity, porosity, degradation, and releasing rates of incorporated biomolecules can be tailored [3, 9]. However, being synthetic, they lack the biochemical information that might be physiologically intrinsic in native tissues [8]. Recently, the use of naturally derived scaffolds has gained attention as they offer a more cost-effective biomimetic alternative to synthetic materials [10]. Such natural scaffolds, with innate bioactive capacities, provide structural and biochemical support for surrounding cells and can be considered a promising cell homing approach that has the ability to enhance cell recruitment, proliferation, release of signalling molecules from the surrounding microenvironment as well as stimulating cell differentiation [3, 11]. Natural endogenous scaffolds in the form of intracanal blood clot or other blood-derivatives have shown many favourable clinical outcomes such as resolution of clinical signs and symptoms, bone healing and continued root development [12–14]. However, several studies have reported unpredictable pattern and architecture of newly formed tissues following the use of the blood clot as a scaffold to revascularize an empty canal [15, 16].

Currently, endodontic literature focuses on the development of scaffolds that can be employed to regulate inflammation and immunologic reactions [11, 17, 18]. This has become emphasized as more studies employing regenerative endodontic procedures for mature permanent necrotic teeth are being published [19–22].

One of the recent alternatives to the blood clot in REPs is the use of decellularized extracellular matrix (dECM)-derived scaffolds [23]. Native tissue extracellular matrix (ECM) is an excellent source for the fabrication of scaffolds due to its ability to mimic the optimal natural environment for tissue regeneration [24]. Extracellular matrix contains structural proteins and growth factors that can orchestrate cellular proliferation, migration and differentiation [25]. Mild decellularization protocols can maintain ECM structure and biocompatibility whilst eliminating the cells and nucleic acids that can cause an immune reaction [24]. Indeed, dECM has been developed as a biologic scaffold for tissue engineering applications in the field of regenerative medicine. Decellularized ECM-derived scaffolds has been shown to facilitate the constructive remodelling of many different tissues in both preclinical animal studies and in human clinical applications [24]. The source from which these scaffold materials is derived includes a variety of tissues, such as heart valves [26], blood vessels [27], skin [28], skeletal muscle [29], ligaments [30], small intestinal submucosa (SIS) [31], urinary bladder [32] and liver [33]. Decellularized ECM-derived scaffolds have shown considerable success when used in vascular grafts [34], skin grafts [35] and in whole-organ regeneration [36].

Hypothetically, providing the dynamic and structural complexity of ECM will result in a microenvironment favourable for lineage specific differentiation of transplanted/recruited stem cells. Recently, decellularized ECM has been investigated as a promising scaffold for regenerative endodontic procedures [23]. However, the clinical feasibility of using decellularized ECM-derived scaffolds in regenerative endodontics is unclear regarding the source of dECM, appropriate decellularization protocol, sterilization and method of scaffold delivery. These unresolved clinical hurdles could further complicate the steps of REPs and increase the cost of treatment. Most importantly, current histological evidence following the use of dECM-derived scaffolds in REPs is scarce [23].

To address these gaps, a systematic review of the literature was conducted aiming to evaluate the role of dECM scaffolds in dentin-pulp regeneration and the potential contributing factors that could influence regenerative outcomes.

## Methodology

### Protocol and registration

The protocol for this review was registered in The International Prospective Register of Systematic Reviews (PROSPERO) as (CRD42023433026) and conducted according to the Preferred Reporting Items for Systematic Reviews and Meta-Analyses Protocol (PRISMA-P) 2020 statement [37].

### Focused question

The research question was formulated as: Can decellularized ECM-derived scaffolds influence the histological outcome of regenerative endodontic applications regarding the nature and pattern of tissues?

- Participants/population: Animal (ectopic, semi-orthotopic or orthotopic) model/ human teeth
- Intervention: Decellularized ECM-derived scaffolds in regenerative endodontics.
- Comparison: non-decellularized ECM-derived scaffolds in regenerative endodontics.
- Outcome: Nature and pattern of regenerated tissues.

### Search strategy

- In June 2023, an electronic search was conducted on PubMed, Scopus, Google Scholar and Web of Science databases as well as a manual search in major endodontic journals (Journal of Endodontics and International Endodontic Journal). The search was updated April, 1st, 2024. The search strategy used a combination of keywords and Medical Subject Heading (MeSH) terms associated with the Boolean operators ‘AND’ and ‘OR’ as shown in Supplementary file 1.
- The pool of studies was further enriched by conducting electronic search in the major endodontic journals, including Journal of Endodontics and International Endodontic Journal to search for articles that were not found in databases.
- Articles retrieved from the search strategy were imported into Endnote X8 software (Thomson Reuters) for duplicate removal.

### Study selection

#### Inclusion criteria

- Original articles published until April 1st, 2023.
- Studies reporting the histological and immunohistochemical results of scaffolds in regenerative endodontic applications.
- Studies in all languages.

#### Exclusion criteria

- Review studies.
- In vitro studies.
- Ex-vivo studies.
- Ongoing trials.
- Studies not including decellularized ECM-based scaffold in their methodology.

The articles identified were screened independently by two reviewers (H.E. and R.E.) for eligibility. Any disagreements were resolved by discussion with a third reviewer (A.A.).

### Data extraction

Data were extracted from eligible articles using pre-designed data extraction tables (Microsoft Word) by two independent reviewers (H.E. and R.E.). Any disagreements were solved by discussion among them. Only the data related to in vivo experiments of the included studies were extracted for analysis. Also, in studies assessing both periodontal tissues and supporting structures regeneration as well as dentin pulp regeneration, data were extracted only for the latter. Extracted data included:

- Characteristics of animal experiments in the included studies: Author, year of publication, animal host, experimental model, sample size, study groups, duration of experiment, evaluation of decellularization and method(s) of histological assessment (Table 1).
- Methods of scaffold characterization, study findings and potential contributing factors to histological outcomes (Table 2).

**Table 1:** Characteristics of animal experiments in the included studies

Author / Year of publication	Animal host	Experimental model	Sample size (n)	Study groups	Duration of study	Evaluation/confirmation of decellularization	Method(s) of histological evaluation (in vivo)
**Ravindran et al., 2014 ** [38]	Nude mice	Subcutaneous implants (ectopic)	Not stated	Two groups: Gp1:ECM + collagen/chitosan Gp2:Collagen/chitosan (control)	Two weeks	-IF analysis (DAPI staining): for detection of nuclei	- Histological (H&E and Alizarin red) - IHC (tubulin, DMP1, DSP, DPP and von Willebrand factor)
**Chen et al., 2015 ** [39]	Nude mice Miniature swine	Subcutaneous implants (ectopic) In jaw-bone (semi-orthotopic)	Not stated *n* = 3/group	Acellular TDM/ECM (in ectopic model) TDMs as control group and APES/TDM/ECM with p-DFSCs as test group (in semi-orthotopic model)	Six weeks for ectopic model Twelve weeks for semi-orthotopic model	-IF analysis (DAPI staining): or detection of nuclei) -MT staining (for detection of optimal decellularization time)	- Histological (H&E and MT) - IHC (periodontal, odontogenic/osteogenic and pulpal markers)
**Zhang et al., 2017 ** [40]	Yucatan mini-pigs	In tooth extraction sockets (semi-orthotopic)	*n* = 48 (*n* = 4/group1a) (*n* = 4/group1b) (*n* = 8/group2a) (*n* = 8/group2b) (*n* = 6/group3a) (*n* = 6/group3b) (*n* = 6/group4a) (*n* = 6/group4b)	Eight groups: -Gp1-a: acellular dTBs: 3 m -Gp1-b: acellular dTBs: 6 m -Gp2-a: recell-dTBs: 3m -Gp2-b: recell-dTBs: 6m -Gp3-a: BMP2-loaded dTBs: 3m -Gp3-b: BMP2-loaded dTBs: 6m -Gp4-a: native TBs (nTBs): 3m -Gp4-b native TBs (nTBs): 6m	Three or six months	-Macroscopic picture -H&E staining -Picrosirius red staining	- Histological (H&E) - IF (DAPI) - IHC (detection of hDPSCs and HUVECs and DSP expression)
**Hu et al., 2017 ** [41]	Nude mice	Subcutaneous implants (semi-orthotopic)	Not stated	-Two groups: Gp1:ECM + hDPSCs in tooth slice Gp2: empty tooth slice (negative control) -Positive control group (not transplanted): tooth slices from healthy wisdom teeth with intact pulp	Eight weeks	- Macroscopic picture of tissues - H&E - DAPI - SEM	- Histological (H&E) - IF DAPI (Col-IV laminin, fibronectin, integrin β1, and vimentin) - IHC for DSPP
**Alqahtani et al., 2018 ** [23]	Beagle dogs	In root canal space (orthotopic)	*n* = 16 Gp1: *n* = 4 Gp2: *n* = 6 Gp3: *n* = 6	Three groups: -Gp1: ECM scaffold -Gp2: collagen scaffold -Gp3: blood clot	Eight weeks	- H&E - DAPI - DNA content	- Histological (Goldner’s trichrome) - IHC (CD31 and DSP)
**Huang et al., 2018 ** [42]	Immunodeficient mice	Subcutaneous implants (semi-orthotopic)	*n* = 24 (*n* = 4/group)	Six groups: -Gp1:plain collagen + hDPSCs -GP2: plain collagen + hBMSCs -Gp3: dual ECM + hDPSCs -Gp4: dual ECM + hBMSCs -Gp5: pulp ECM + hDPSCs -Gp6: pulp ECM + hBMSCs	Four weeks	Not stated	- Histological (H&E) - IHC (DSP, DPP DMP-1 and VEGF)
**Bakhtiar et al., 2020 ** [43]	Sprague–Dawley rats	Subcutaneous implants (ectopic)	*n* = 24 (*n* = 4/group)	Six groups: 1.5, 2.25 & 3.00 mg/ml concentrations of cross-linked scaffolds and 1.5, 2.25 & 3.00 mg/ml concentrations non cross-linked scaffolds)	Two weeks	- DNA quantification - Histological evaluation (MT, AB and H&E)	- Histological (H&E, MT and Toluidine blue) - IHC (CD68 and SMA)
**Bakhtiar et al., 2021 ** [44]	Sprague–Dawley rats	Subcutaneous implants (ectopic)	Not stated	dECM of protocol #7 was subcutaneously implanted	Two weeks	- DNA quantification - Histological evaluation (MT, Safranin O and H&E)	- Histological (H&E and MT)
**Alghutaimel et al., 2021 ** [45]	Severely combined immunodeficient (SCID) mice	Subcutaneous implants (semi-orthotopic)	*n* = 12 *n* = 4/ group	Three groups: Gp1:dECM + hDPSCs, Gp2:dECM unseeded, Gp3: empty root slice “control”	Four weeks	- DNA quantification - H&E staining - DAPI staining - SEM	- Histological (H&E) - IHC (for detection of human nuclei)
**Tan et al., 2021 ** [46]	Nude mice	Subcutaneous implants (ectopic)	*n* = 20/group (*n* = 5 sites/mouse/group)	Five groups: (Gp1:PBS + cells, Gp2:green fluorescent protein + cells, Gp3:dECM + cells, Gp4: BMP4 + cells and Gp5 dECM + BMP4 + cells)	Four weeks	- H&E staining - DAPI staining	- Histological (H&E)
**Fu et al., 2021 ** [47]	Beagle dogs	In jaw-bone (semi-orthotopic)	*n* = 6 (*n* = 3/ group)	Two groups: Gp1: laminin coated dECM + TDM, Gp2: dECM + TDM	12 weeks	- Gross morphology - DAPI	- Histological (H&E) - IHC (DSPP, Col-I, laminin and DMP-1) compared to native pulp
**Kim et al., 2021 ** [48]	Nude mice	Subcutaneous implants (semiorthotopic)	*n* = 20/ group	- Gp1: dPDL-ECM + hPDLSs - Gp2: dP-ECM + hDPSCs - Gp3: dPDL-ECM (control) - Gp4: dP-ECM (control)	9 weeks	- Not stated	- Histological (H&E and MT) - IHC (CP23, OC, VEGF, CD34, HN, Col-XII and DSP) for 6 samples/group
**Bakhtiar et al., 2022 ** [49]	Sprague–Dawley rats	Subcutaneous implants (ectopic) Subcutaneous implants (semi-orthotopic)	*n* = 3/group *n* = 12 (*n* = 6 per group)	One group: Freeze-dried sponges (for immunogenicity) Root segments filled with cell-free or cell-loaded HAM scaffolds implanted in rats’ calvaria subcutaneous space	2 weeks 7 weeks	- DNA content evaluation - Histological evaluation (MT and H&E)	- Histological (H&E and MT) - Histological (H&E and MT) - IHC: Col-I
**Zheng et al., 2023 ** [50]	Immunodeficient nude mice	Subcutaneous implants (semi-orthotopic)	*n* = 3	Gp1: tooth slices filled with cell-seeded dECM/GelMA microspheres Gp2: tooth slices filled with cell-seeded GelMA microspheres Gp3: tooth slices filled with cells	12 weeks	- H&E - DAPI - Picrosirius Red (for collagen) - AB (for GAGs)	- Histological (H&E and MT) - IHC (CD31 and DSPP) - Calcein fluorescent labelling for newly formed dentin
**Shi et al.,** **2023 ** [51]	Immunodeficient mice	Subcutaneous implants (semi-orthotopic)	*n* = 12 (*n* = 6 per group)	Gp1: treated tooth slices filled with recellularized DSMG Gp2: treated tooth slices filled with acellular DSMG	12 weeks	- Histological evaluation (H&E and MT)	- Histological (H&E and Sirius red) - IHC (CD31 and DSPP)
**Bakhtiar et al.** [52]**, 2023**	Sprague–Dawley rats	Subcutaneous implants (ectopic) Subcutaneous implants (semi-orthotopic)	*n* = 18 (*n* = 6 per group) *n* = 24 (*n* = 6 per group)	Three groups: Gp1: 22.5 mg/ml crosslinked, Gp2: 30 mg/ml crosslinked, Gp3: 30 mg/ml not crosslinked Four groups with root segments filled with either: Gp1: 22.5 mg/ml + cells, Gp2: 30 mg/ml + cells, Gp3: 22.5 mg/ml, cell free, Gp4: 30 mg/ml, cell free. All were crosslinked gels	2 weeks 6 weeks	- Histological evaluation (MT and H&E)	- Histological (H&E) - Histological (H&E and MT) - IHC: Col-I, CD31
**Yuan et al.** [53]**, 2023**	Nude mice	Subcutaneous implants (semi-orthotopic)	*n* = 40 (*n* = 10 per group)	Four groups; all cell-loaded scaffolds, Gp1: 5 mg/ml dECM + TDM tube, Gp2: 7.5 mg/ml dECM + TDM tube, Gp3: 10 mg/ml dECM = TDM tube, Gp4: GelMA hydrogel + TDM tube	8 weeks	- DNA content evaluation - H&E - MT - Safranin O - DAPI - Collagen and GAGs quantification	- Histological (H&E and MT) - IF: anti-DSPP and anti-mitochondria

*Abbreviations*: *AB* Alcian blue, *ALP* Alkaline phosphatase, *APES* Aligned Poly(D,Llactide-co-glycolide)/gelatin electrospun sheet, *bFGF* Basic fibroblast growth factor, *BMP-2* Bone morphogenetic protein-2, *BMP-4* Bone morphogenetic protein-4, *CD31* Cluster of differentiation-31, *CD34* Cluster of differentiation-34, *CD68* Cluster of differentiation-68, *Col* Collagen, *CP23* Cementum-derived protein 23, *DAPI* 4',6-diamidino-2-phenylindole, *dECM* Decellularized extracellular matrix, *dp-ECM* Decellularized pulp extracellular matrix, *dPDL-ECM* Decellularized periodontal ligament extracellular matrix, *DPP* Dentin phosphoprotein, *DSMG* Decellularized submandibular gland, *DSP* Dentin sialoprotein, *DSPP* Dentin sialophospho protein, *DMP-1* Dentin matric protein 1, *dTBs* Decellularized tooth buds, *FN* Fibronectin, *GAGs* glycosaminoglycans, *GelMA* Gelatine methacrylate, *HAM* Human amniotic membrane, *hBMSCs* Human bone marrow stem cells, *hDPSCs* Human dental pulp stem cells, *H&E* Hematoxylin and eosin, *HUVECs* Human umbilical vein endothelial cells, *HN* Human nuclei, *IF* Immunofluorescence, *IHC* Immunohistochemistry, *LN* Laminin, *MMP* Matrix metalloproteinase, *MT* Masson’s trichrome, *nTB* Native tooth bud, *OC* Osteocalcin, *PBS* Phosphate buffered saline, *Recell-dTB* Recellularized dental tooth bud, *SDS* Sodium dodecyl sulphate, *SEM* Scanning electron microscope, *SMA* Smooth muscle actin, *TBs* Tooth buds, *TDM* Treated dentin matrix, *TGFβ* Transforming growth factor beta, *VEGF* Vascular endothelial growth factor, *vWF* Von Willebrand factor

**Table 2:** Scaffold characterization and potential contributing factors to histological outcomes

Author / Year of publication	Methods of characterization of ECM scaffold	Potential contributing factors						Histological outcomes
		**Source of ECM**	**Method of decellularization**	**Sterilization of scaffold**	**Form of scaffold delivery and crosslinking**	**Concentration of ECM components**	**Source of cells (cell homing or cell transplantation)**	
**Ravindran et al., 2014 ** [38]	-RT-qPCR: In vitro differentiation of DPSCs (with or without DPP) and PDLSCs on ECM scaffold -IHC (tubulin, FN, BMP-2, TGFβ, VEGF, MMP2, MMP9, phospho serine and phosphor tyrosine, DMP1, DSP, DPP, thrombospondin and von Willebrand factor)	DPSCs-generated ECM	-DPSCs cultured in collagen/chitosan hydrogel for 2weeks -Cell lysis -DNase treatment	Not stated	ECM-embedded collagen/chitosan scaffold/no crosslinking	Not stated	Cell homing	- Neovascularization - Dental pulp-like tissue - Cells highly expressing DSP and DPP -Increased calcium deposition and polarization of collagen fibrils
**Chen et al., 2015 ** [39]	-Gross anatomy of dECM -SEM: for ultrastructure analysis -IHC: for ECM proteins (Col-I, Col-III, FN and LN) -Cell based analysis	Miniature swine dental pulp	-1% SDS for 12 h -Triton X-100 for 30 m -PBS washes	Immersion in a solution containing penicillin/streptomycin for 48 h	dECM placed in pulp cavity of TDM for ectopic model/ APES + TDM + dECM composite for orthotopic model (no crosslinking)	Not stated	Cell transplantation (Porcine dental follicle stem cells (pDFSCs))	For ectopic model: -Vascularized tissue positive for LN and CD31 For semi-orthotopic model: -Dental pulp-like tissues positive for Col-1, Col-III, DMP-1 and DSPP -Cellular distribution similar to native odontoblastic layer -Pre-dentine matrix-like deposition on the interface between ECM and TDM
**Zhang et al., 2017 ** [40]	-Picrosirius red staining: for collagen matrix evaluation -IF analysis (Vimentin, E-cadherin and Factor VIII): to confirm the survival of seeded cells prior to scaffold implantation	Porcine tooth buds	-1% SDS 24 h -1%TritonX-100 24h -Nuclease treatment 3 h -10% EDTA decalcification 3 months	Not stated	Decellularized enamel organ + pulp organ construct / (no crosslinking)	Not stated	Cell transplantation (hDPCs and HUVECs) vs cell homing (acellular scaffold)	-Well-organized dentin (positive for DSP) and high pulp cellularity similar to that of natural dental pulp in recell-dTB groups -dTB constructs appeared cellularized but formed less organized dentin
**Hu et al., 2017 ** [41]	-Microstructure evaluation by SEM -IF staining for (Col-IV, laminin, fibronectin, integrin β1, and vimentin)	Miniature swine dental pulp	-10% SDS on shaker for 32 h -Deionized water on shaker for 4 h 1%Triton X-100 on shaker for 2 h -DNase/ RNase for 1 hPBS wash for 2 h	Placed in PBS containing streptomycin,penicillin G, and amphotericin B for 12 h	Decellularized tissues sized to fit the lumen of tooth slices (no crosslinking)	Not stated	Cell transplantation (hDPSCs)	-Pulp-like fibrous vascularized tissues in ECM group -A layer of newly formed, mineralized tissue lined by a layer of odontoblast-like cells -Calcific deposits showed high expression of DSPP
**Alqahtani et al., 2018 ** [23]	-Proliferation and migration of DPSCs on digested ECM -IF staining for (Col-I, DSP, DMP-1 and vWF) -SEM -Growth factors quantification by ELISA (VEGF, bFGF and TGF-β1)	Swine dental pulp	-Trypsin/EDTA for 1h -3% TX-100 30 m -4% deoxycholic acid 30 m -0.1% PAA in 4% ethanol for 15 m then PBS wash for 48 h (all steps in vacuum incubator)	-Immersion in peracetic acid (for in vitro) -EtO after lyophilization (for in vivo)	Lyophilized sheets/(no crosslinking)	100 mg per canal	Cell homing	-All 3 groups showed evidence of intracanal mineralization -CD31-positive cells and DSP-positive Canine tissues in the pulp canals in ECM group
**Huang et al., 2018 ** [42]	-IHC: (FN, DMP-1, DPP, DSP, TGF-β1, BMP-2, vWF, VEGF and bFGF) compared to pulp ECM (control) -Cell based analysis	hDPSCs and HUVECs-generated ECM	-DPSCs cultured in collagen/chitosan hydrogel for 2 weeks -Cell lysis (ammonium hydroxide) -DNase treatment -Same scaffold seeded with HUVECs then decellularization protocol was repeated	Not stated	Lyophilized pulp-ECM or dual-ECM scaffolds placed within canal space of TDM (human tooth slices) /(no crosslinking)	Not stated	Cell transplantation with either hDPSCs or HMSCs	-Pulp-like tissue in both dual-ECM and pulp-ECM groups -More robust vascularization in dual-ECM group -No significant difference between the two scaffolds regarding the expression of odontogenic proteins and proangiogenic proteins (both significantly higher than control)
**Bakhtiar et al., 2020 ** [43]	-Pore size analysis by SEM -Porosity percentage -PBS absorption -Degradation rate -Cell based analysis:	Bovine dental pulp	-Trypsin and EDTA on shaker for 1h -SDS 48h -DNase treatment -PBS washes	Not stated	Lyophilized hydrogel sponges -chemically crosslinked	1.50, 2.25 or 3.00 mg/ml	Cell homing	-Neovascularization in all groups -More angiogenesis was observed in higher concentration of crosslinked pulp ECM -New bone-like tissue in cross-linked 2.25 mg/ml and 3.00 mg/ml groups -Mononuclear macrophage (CD68) infiltration was the least in the cross-linked 3.00 mg/ml group
**Bakhtiar et al., 2021 ** [44]	- DAPI for protocol#7 samples - IF (Col-I)	Bovine dental pulp	Trypsin/EDTA and /or SDS treatment for variable durations followed by DNase treatment for 1 h	Not stated	Lyophilized sheets/ (no crosslinking)	Not stated	Cell homing	-Scaffolds gradually degraded and replaced by highly vascularized connective tissues and fibrous encapsulation -Macrophages, lymphocytes and other chronic inflammatory cells were evident
**Alghutaimel et al., 2021 ** [45]	- H&E after recellularization by DPSCs (at 7, 14 and 21 days) - IHC (VEGFA, FGF-2 and CD31) - SEM - Fluorescent staining and confocal scanning laser microscopy for the recellularized ECM - RT-qPCR for the recellularized ECM	Bovine dental pulp (incisors)	-Freeze–thaw cycle -Hypotonic Tris buffer (containing EDTA and aprotinin) for 16 h -Hypotonic Tris–HCl buffer (containing SDS, EDTA and aprotinin) for 24 h -Tris–HCl (containing magnesium chloride, bovine serum albumin, DNase and RNase) for 3 h	Immersion in 0.1% (v/v) peracetic acid at room temperature for 3 h	dECM (cell-seeded or unseeded) placed within pulp space of human root slices/ (no crosslinking)	Not stated	Cell transplantation (hDPSCs) vs cell homing	-Seeded dECM group showed cellular organization pattern (positive for human nuclei) resembling that of the native dental pulp -Unseeded dECM group showed host cell migration and repopulation
**Tan et al., 2021 ** [46]	- SEM - Alkaline phosphatase (ALP) and Alizarin Red staining of DPSCs - RT‑qPCR (odontogenic/osteogenic and angiogenic genes)	Human dental pulp from 3rd molars	10% SDS on a shaker for 24 h, 1% Triton X-100 for 24 h. then PBS wash for 2 h	Tissues were placed in PBS containing streptomycin and penicillin for 12 h	Powder resuspended in PBS with the addition of BMP4 and/or hDPSCs/ (no crosslinking)	Not stated	Cell transplantation (hDPSCs)	-Pulp-like tissue in the dECM + BMP-4 + DPSC group -No pulp-like tissue in the control group and in test groups not containing BMP-4 -No dentin-like tissue was formed in any of the groups
**Fu et al., 2021 ** [47]	-IF analysis for detection of laminin before and after cell seeding -SEM before and after cell seeding	Swine dental pulp	-1% SDS 12 h -1% Triton X-100 30 m -PBS wash 30 m	Immersion in a solution containing penicillin/stretomycin for 48 h	dECM tissue/LN/TDM or dECM tissue /TDM (no crosslinking)	Not stated	Cell homing	-Cementum/bone-like structures and blood vessels were regenerated in the dECM-alone group -Odontoblastic layer-like structures were observed on the interface between dental pulp–like tissues and the dentin matrix in the dECM/LN group (positive for Col-I, DSPP, DMP-1 and LN)
**Kim et al., 2021 ** [48]	No characterization	Tooth slices + Human dental pulp + human periodontal ligament	Tooth slices were incubated in 1% Triton X-100 for 24 h and then 1% SDS for 24 h (cycle repeated 3 times on shaker)	Not stated	Whole dECM tissue + tooth slices (no crosslinking)	Not stated	Cell transplantation (hDPSCs) versus cell homing	-Hard tissue formation was observed positive for DSP and OC -Less hard tissue formation in cell-free group
**Bakhtiar et al., 2022 ** [49]	-Pore size analysis by SEM -Porosity percentage -PBS absorption -Degradation rate -Cell based analysis:	Human amniotic membrane (HAM)	Trypsin and EDTA on shaker for 2 h PBS washes	No sterilization was stated Tissues were only washed with saline containing penicillin/ streptomycin and amphotericin B (before decellularization)	Lyophilized hydrogel sponges -chemically crosslinked	15, 22.5 or 30 mg/ml (Only the 30 mg/ml conc was used in vivo)	Cell-free in immunogenicity experiment Cell-free versus cell-loaded (hDPSCs) groups in pulp regeneration experiment	-Immunogenicity experiment: neovascularization and mild-moderate inflammatory response -Pulp regeneration experiment: scaffolds were replaced by highly vascularized pulp-like tissues with high collagen content with no statistically significant difference between groups
**Zheng et al., 2023 ** [50]	-Degradation rate -Cytocompatibility (live/dead assay and DAPI) -IF (Ki67, DSPP and *β*- III tubulin) -RT‑qPCR (odontogenic genes: RUNX2, DSPP and DMP-1 and for angiogenic genes: CD31, VEGF and for neurogenic gene: nestin)	Human dental pulp	1% Triton X-100 for 24 h 1% SDS for 24 h then replaced every 24 h for 3 cycles (all on shaker)	The powder was sterilized with ethylene oxide	Hydrogel form of dECM modified-GelMA microspheres/(crosslinked)	10 mg/ml before crosslinking	Cell transplantation (hDPSCs)	-Vascularized pulp-like tissue, a layer of odontoblast-like cells and newly formed dentin-like tissue in dECM/GelMA group (highly expressed DSPP and CD31) -New dentin formation in GelMA group with less vascularized soft tissue -No hard tissue formation in (cells only) group with loose soft tissue formation
**Shi et al.,** **2023** [51]	- H&E - IF (Col-I, Col-III and FN) - MT and sirius red staining for collagen content - SEM - Cell based analysis:	Rat submandibular gland	10% SDS for 32 h, washed with deionized water, then 1% Triton X-100 for 2 h then PBS wash	Immersion in Penicillin/streptomycin and amphotericin B for 12 h	Lyophilized sheets/ (no crosslinking)	Not stated	Cell-free versus cell-loaded (hDPSCs) groups	-New soft and hard tissue formation in both groups -Higher number of perfused and CD31 positive blood vessels -Highly organized pulp tissue, odontoblast-like layer and newly secreted dentin in recellularized group -Tissues were positive for DSPP only in recellularized group
**Bakhtiar et al.** [52]**, 2023**	- Degradation - SEM - Rheological analysis of the hydrogel - Cell based analysis	Human amniotic membrane (HAM)	Trypsin and EDTA and placed on shaker for 2 h Then PBS washes	Not stated	Hydrogel form/(chemically crosslinked)	22.5 or 30 mg/ml	Cell free hydrogels for immunogenicity experiment Cell-free versus cell-loaded (hDPSCs) groups for pulp regeneration experiment:	-Immunogenicity experiment: neovascularization in all groups and the fibrous capsule’s thickness was the same in all groups -Pulp regeneration experiment: Quality of pulp-like tissue in cell- loaded hydrogels appeared to be less optimal than the cell-free hydrogels Highest concentration resulted in highly vascularized pulp-like tissue
**Yuan et al.** [53]**, 2023**	- SEM - Rheological analysis of the hydrogel - Swelling and degradation - Cell based analysis	Swine dental pulp	Trypsin and EDTA for 6 h, 12 h or 18 h,,TX-100 for 3 h, then DNase treatment for 24 h	Immersion in Penicillin/streptomycin for 24 h	Hydrogel form (no crosslinking)	5, 7.5 or 10 mg/ml	Cell-loaded (hDPSCs)	-H&E and Masson’s staining showed pulp-like tissues in all dECM hydrogel groups exhibited abundant neovascularization throughout the root canals (more in 10 mg/ml concentration group) - Odontoblast-like layer detected by IF in dECM groups (7.5 and 10 mg/ml) -GelMA group displayed neovascularization solely at both ends of the root canals adjacent to the host tissue

*Abbreviations*: *ALP* Alkaline phosphatase, *bFGF* Basic fibroblast growth factor, *BMP-2* Bone morphogenetic protein-2, *BMP-4* Bone morphogenetic protein-4, *CD31* Cluster of differentiation-31, *CD68* Cluster of differentiation-68, *Col* Collagen, *DAPI* 4',6-diamidino-2-phenylindole, *dECM* decellularized extracellular matrix, *DSMG* decellularized submandibular gland, *DPP* Dentin phosphoprotein, *DSP* Dentin sialoprotein, *DSPP* Dentin sialophospho protein, *DMP-1* Dentin matric protein 1, *dTBs* decellularized tooth buds, *EDTA* Ethylenediamine tetraacetic acid, *EtO* Ethylene oxide, *FN* Fibronectin, *GAGs* Glycosaminoglycans, *GelMA* Gelatine methacrylate, *HAM* human amniotic membrane, *hBMSCs* Human bone marrow stem cells, *hDFSCs* human dental follicle stem cells, *hDPCs* human dental pulp cells *hDPSCs* human dental pulp stem cells, *HUVECs* Human umbilical vein endothelial cells, *IF* Immunofluorescence, *Ki67* marker of proliferation, *LN* Laminin, MMP: matrix metalloproteinase, *MSCs* Mesenchymal stem cells, *MTT assay* 3-(4,5-dimethylthiazol-2-yl)-2,5-diphenyl-2H-tetrazolium bromide, *OC* Osteocalcin, *PBS* Phosphate buffered saline, *pDFSCs* porcine dental follicle stem cells, *Recell-dTB* Recellularized dental tooth bud, *RT-qPCR* Reverse transcriptase quantitative polymerase chain reaction,, *RUNX-2* Runt-related transcription factor 2, *SDS* sodium dodecyl sulphate, *TDM* Treated dentin matrix, *TGFβ* Transforming growth factor beta, *TX-100* Triton X-100, *VEGF* Vascular endothelial growth factor, *vWF* Von Willebrand factor

List of abbreviations mentioned in the tables is included in supplementary file 6.

### Reporting quality assessment [54]

Assessment of the reporting quality for all studies included in this systematic review was performed using the Animal Research: Reporting of In Vivo Experiments (ARRIVE) guidelines [54] (H.E. and R.E.). In order to evaluate the 21 items, a modified scoring system [55] was used as follows: all subitems were reported “Yes” = 2 points, not all subitems were reported “Unclear (Uc)” = 1 point, or all subitems were not reported “No” = 0 points. Afterwards, quality coefficient (QC) was generated by calculating the sum of all the points obtained for each study divided by 42 “the maximum possible points per study”. The quality of study was reported as “Excellent” when QC was 0.8–1, “Average” if QC was 0.5–0.8 and “Poor” if QC was < 0.5.

### Risk of bias assessment [56]

Assessment of bias for all studies included in this systematic review was performed using SYstematic Review Centre for Laboratory animal Experimentation (SYRCLE) risk of bias (RoB) tool [56] (H.E. and R.E.). A modified scoring system [57] was used according to the total number of “yes” answers to the assigned questions (Yes = 1 point) The degree of bias was calculated as follows: High risk: 0–4, Moderate risk: 5–7 and Low risk: 8–10.

## Results

### Study selection

**Fig. 1:**
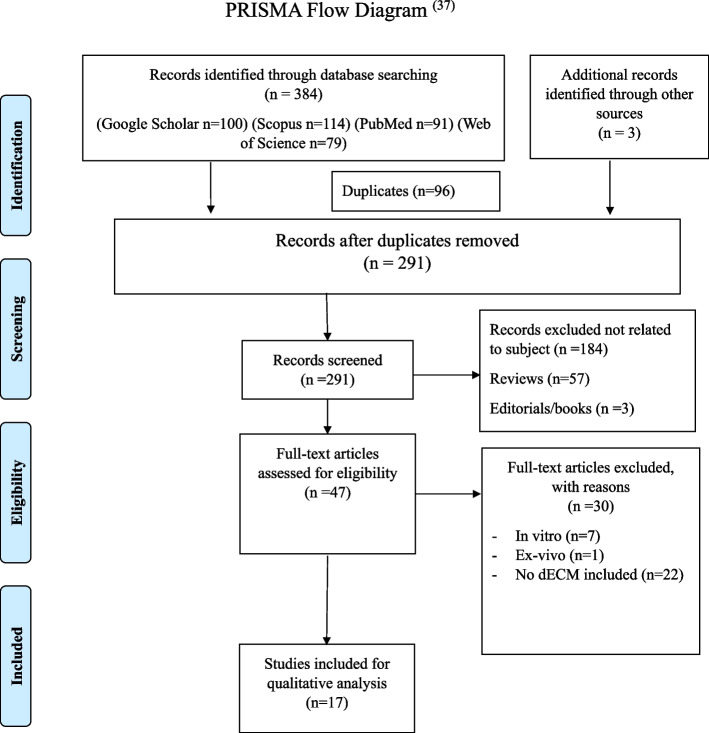
PRISMA flow diagram showing inclusion and screening process of included records

The records from the different databases were combined (*n* = 384) then duplicates were removed (*n* = 96). Then additional records identified through other sources were added (*n* = 3). For the initial screening of the imported records (*n* = 291), the titles of the papers were identified and decided whether they were relevant to the topic or not (*n* = 184). Reviews were excluded (*n* = 57) and editorials/books were excluded (*n* = 3). For the final screening, the abstracts of all relevant articles (*n* = 47) were then carefully appraised to identify eligible studies. Full texts of the relevant articles were screened. During this final screening phase, studies were excluded as: in vitro studies (*n* = 7), ex-vivo studies (*n* = 1), and studies not including dECM (*n* = 22). Excluded studies by full text screening (*n* = 30) and reasons for exclusion are listed in supplementary file 2. Total number of studies included in this systematic review was 17 studies (*n* = 17). The flow chart for search process and study inclusion are presented in (Fig. 1) according to PRISMA 2020 guidelines [37].

### Study characteristics

Different animal models were used in the included studies. The majority used mice [38, 39, 41, 42, 45, 46, 48, 50, 51, 53] followed by rats [43, 44, 49, 52], pigs [39, 40] and beagle dogs [23, 47]. The main source of ECM used was swine [23, 39, 41, 47, 53], human [46, 48, 50] or bovine dental pulp [43–45]. Others used cell-generated ECM [38, 42], swine tooth bud [40], human amniotic membrane [49, 52] and rats’ submandibular glands [51]. Regarding the study model, scaffolds were ectopically transplanted in six studies [38, 39, 43, 44, 46, 52], semi-orthotopically transplanted in twelve studies [39–42, 45, 47–53] and orthotopically transplanted in just one study [23]. In terms of the regenerative approach, two thirds of the included studies used cell-seeded scaffolds [39–42, 45, 46, 48–53] while the rest used cell-free scaffolds [23, 38, 43, 44, 47]. Six of the former studies also compared between the two approaches [40, 45, 48, 49, 51, 52]. As for the scaffold form, this was either tissue sheets placed within either treated dentin matrix TDM [39, 42, 47, 51, 53] /tooth slices [41, 45, 48], or freeze-dried sponges [43, 49] or sheets [23, 44, 51], powder [46], hydrogel microspheres [50], injectable hydrogel [52, 53] or whole decellularized tooth bud [40]. Only four studies used crosslinked scaffolds [43, 49, 50, 52]. Regarding terminal sterilization, scaffolds were either immersed in penicillin/streptomycin [39, 41, 46, 47, 51, 53], or sterilized using ethylene oxide (EtO] [23, 50] or peracetic acid (PAA) [45]. The rest of studies did not report the method of sterilization [38, 40, 42–44, 48, 49, 52]. Interestingly, the concentration of ECM components was reported by only six studies [23, 43, 49, 50, 52, 53]^.^ Regarding the methods of in vivo outcome assessment, only histological evaluation was performed in two studies [44, 46], while histological and immunohistochemical analysis were done in the rest of the studies. When ECM scaffolds were compared to other non-ECM-derived scaffolds, regeneration of both pulp-like and dentin-like tissues were reported in the ECM groups in eight studies [23, 38–42, 47, 50] while pulp-like tissue was solely reported in five studies [44, 46, 49, 52, 53]. Regarding the cell homing approach versus cell transplantation approach, it was noted that in cell-seeded scaffolds, pulp-like and dentin-like tissue regeneration was more likely to be detected compared to cell-free scaffolds [39–42, 46, 48, 50, 51]. Conversely, quality of pulp-like tissues in cell-seeded group was reported, in one study, to be less optimal that in cell-free groups [52].

### Reporting quality assessment

All studies were analysed using Animal Research: Reporting of In Vivo Experiments (ARRIVE) guidelines. Study design was described by all studies [23, 38–53]. However, none of the studies provided precalculated sample size [23, 38–53]. Inclusion and exclusion criteria were not properly described in any of the studies [23, 38–53]. Randomization was carried out in only three studies [45, 49, 52]. Blinding was only carried out in four studies [43–45, 52]. Outcome measures and experimental animals’ details were adequately described by all studies [23, 38–53]. Statistical analysis of in vivo outcomes was not performed in ten studies [23, 28, 38, 40, 42, 44, 46, 47, 52, 53] Experimental steps were unclear in all studies [23, 38–53]. Results were adequately described with statistical evaluation of each group in only five studies [41, 43, 45, 48, 51]. Data on the animal model and in vivo outcomes were properly described in abstracts in only seven studies [23, 39, 40, 45–47, 52, 53]. The introduction section provided adequate background on the topic in all studies [23, 38–53], however, none of them explained the rationale of using a specific animal model and its relevance to human biology. In all studies [23, 38–53], objectives were clearly described. In one study [44], the ethical statement was not provided. Housing details and animal care were reported in only three studies [23, 40, 41]. Adequate interpretation of results as well as study protocol registration were done in all studies [23, 38–53]. In one study [38], the generalizability of outcomes and clinical implications of experimental results were not stated. Data access statement was mentioned in only six studies [23, 47, 48, 50, 51, 53]. While declaration of interest statement was mentioned in all studies except for two [38, 39]. Overall, quality coefficient ranged between 0.5–0.7 indicating an “Average” grade of all the reviewed studies. The assessment criteria and their results are listed in Table 3.

**Table 3:** Quality assessment of included studies using Animal Research: Reporting of In Vivo Experiments (ARRIVE) guidelines

**Reference**	**Ravindran et al. ** [38]	**Chen et al. ** [39]	**Zhang et al. ** [40]	**Hu et al. ** [41]	**Alqahtani et al. ** [23]	**Huang et al. ** [42]	**Bakhtiar et al. ** [43]	**Bakhtiar et al. ** [44]	**Alghutaimel et al. ** [45]	**Tan et al. ** [46]	**Fu et al. ** [47]	**Kim et al. ** [48]	**Bakhtiar et al. ** [49]	**Zheng et al. ** [50]	**Shi et al. ** [51]	**Bakhtiar et al.** [52]	**Yuan et al.** [53]
**Criteria of evaluation**																	
**Essential 10**																	
1. Study design described	Yes	Yes	Yes	Yes	Yes	Yes	Yes	Yes	Yes	Yes	Yes	Yes	Yes	Yes	Yes	Yes	Yes
2. Precalculated sample size	No	No	No	No	No	No	No	No	No	No	No	No	No	No	No	No	No
3. Inclusion and exclusion criteria	Uc	Uc	Uc	Uc	Uc	Uc	Uc	Uc	Uc	Uc	Uc	Uc	Uc	Uc	Uc	Uc	Uc
4. Randomization of samples into groups	No	No	Uc	No	No	No	No	No	Yes	No	No	No	Yes	No	No	Yes	No
5. Blinding during evaluation	No	No	No	No	No	No	Yes	Yes	Yes	No	No	No	No	No	No	Yes	No
6. Outcome measures described	Yes	Yes	Yes	Yes	Yes	Yes	Yes	Yes	Yes	Yes	Yes	Yes	Yes	Yes	Yes	Yes	Yes
7. Statistical analysis done	No	No	No	Yes	No	No	Yes	No	Yes	No	No	Yes	Yes	Yes	Yes	Yes	Yes
8. Experimental animal details described	Yes	Yes	Yes	Yes	Yes	Yes	Yes	Yes	Yes	Yes	Yes	Yes	Yes	Yes	Yes	Yes	Yes
9. Adequate experimental steps described	Un	Uc	Uc	Uc	Uc	Uc	Uc	Uc	Uc	Uc	Uc	Uc	Uc	Uc	Uc	Uc	Uc
10. Results with descriptive statistics for each group	Uc	Uc	Uc	Yes	Uc	Uc	Yes	Uc	Yes	Uc	Uc	Yes	Uc	Uc	Yes	Yes	Yes
**Recommended set**																	
11. Detailed abstract	Uc	Yes	Yes	Uc	Yes	Uc	Uc	Uc	Yes	Yes	Yes	Uc	Uc	Uc	Uc	Uc	Uc
12. Adequate background	Uc	Uc	Uc	Uc	Uc	Uc	Uc	Uc	Uc	Uc	Uc	Uc	Uc	Uc	Uc	Uc	Uc
13. Objectives/hypotheses adequately described	Yes	Yes	Yes	Yes	Yes	Yes	Yes	Yes	Yes	Yes	Yes	Yes	Yes	Yes	Yes	Yes	Yes
14. Ethical statement	Yes	Yes	Yes	Yes	Yes	Yes	Yes	No	Yes	Yes	Yes	Yes	Yes	Yes	Yes	Yes	Yes
15. Housing details	No	No	Yes	Yes	Yes	No	No	No	No	No	No	No	No	No	No	No	No
16. Animal care and monitoring described	No	No	Uc	Uc	Yes	No	No	No	No	No	No	No	No	No	No	No	No
17. Adequate interpretation of results	Yes	Yes	Yes	Yes	Yes	Yes	Yes	Yes	Yes	Yes	Yes	Yes	Yes	Yes	Yes	Yes	Yes
18. Comments on generalizability and possible clinical translation	No	Yes	Yes	Yes	Yes	Yes	Yes	Yes	Yes	Yes	Yes	Yes	Yes	Yes	Yes	Yes	Yes
19. Protocol registration	Yes	Yes	Yes	Yes	Yes	Yes	Yes	Yes	Yes	Yes	Yes	Yes	Yes	Yes	Yes	Yes	Yes
20. Data access statement	No	Uc	No	No	Yes	No	No	No	No	No	Yes	Yes	No	Yes	Yes	No	Yes
21. Declaration of interest statement	No	No	Yes	Yes	Yes	Yes	Yes	Yes	Yes	Yes	Yes	Yes	Yes	Yes	Yes	Yes	Yes
**Score**	19	23	28	29	30	23	28	23	31	24	26	28	27	27	28	30	28
**Quality coefficient QC**	0.5	0.5	0.7	0.7	0.7	0.5	0.7	0.5	0.7	0.6	0.6	0.7	0.6	0.6	0.7	0.7	0.7
**Quality grade**	**Average**																

### Risk of bias (RoB) assessment

All studies were analysed using SYstematic Review Centre for Laboratory animal Experimentation (SYRCLE) risk of bias (RoB) tool [56]. Regarding selection bias, in all studies, allocation sequence was unclear [23, 38–53]. However, baseline characteristics of animals were similar among different groups in all studies [23, 38–53]. Only three studies reported concealment of sample allocation [40, 49, 52]. Investigators were blinded in only two studies [44, 45]. Outcome assessors were blinded in four studies [44, 45, 49, 52]. Random allocation of animals in study groups, random selection of animals for assessment, and addressing incomplete outcome data, were mentioned in none of the included studies [23, 38–53]. Regarding reporting bias, there was no selective reporting of outcomes in any of the studies [23, 38–53]. All of the reviewed studies showed high risk of bias where twelve studies scored 2/10 in RoB assessment [23, 38–43, 46–48, 50–53] while the remaining three studies [44, 45, 49] scored 4/10. The overall risk of bias was high in all studies. The risk of bias assessment criteria and their results are listed in Table 4.

**Table 4:** Risk of bias assessment of included studies using SYRCLE risk of bias tool for animal studies

Study (1st Author, Year)	Selection bias	Selection bias	Selection bias	Performance Bias	Performance Bias	Detection Bias	Detection Bias	Attrition bias	Reporting Bias	Other	Score /10	Risk of bias
	**1. Was the allocation sequence adequately generated & applied?**	**2. Were the groups similar at baseline or were they adjusted for confounders in the analysis?**	**3. Was the allocation adequately concealed?**	**4. Were the animals randomly housed during the experiment?**	**5. Were the caregivers and/or investigators blinded from knowledge which intervention each animal received during the experiment?**	**6. Were animals selected at random for outcome assessment?**	**7. Was the outcome assessor blinded?**	**8. Were incomplete outcome data adequately addressed?**	**9. Are reports of the study free of selective outcome reporting?**	**10. Was the study apparently free of other problems that could result in high risk of bias?**		
Ravindran et al., 2014 [38]	Uc	Yes	Uc	Uc	Uc	Uc	Uc	Uc	Yes	Uc	2	High
Chen et al., 2015 [39]	Uc	Yes	Uc	Uc	Uc	Uc	Uc	Uc	Yes	Uc	2	High
Zhang et al., 2017 [40]	Uc	Yes	Yes	Uc	Uc	Uc	Uc	Uc	Yes	Uc	3	High
Hu et al., 2017 [41]	Uc	Yes	Uc	Uc	Uc	Uc	Uc	Uc	Yes	Uc	2	High
Alqahtani et al., 2018 [23]	Uc	Yes	Uc	Uc	Uc	Uc	Uc	Uc	Yes	Uc	2	High
Huang et al., 2018 [42]	Uc	Yes	Uc	Uc	Uc	Uc	Uc	Uc	Yes	Uc	2	High
Bakhtiar et al., 2020 [43]	Uc	Yes	Uc	Uc	Uc	Uc	Uc	Uc	Yes	Uc	2	High
Bakhtiar et al., 2021 [44]	Uc	Yes	Uc	Uc	Yes	Uc	Yes	Uc	Yes	Uc	4	High
Alghutaimel et al., 2021 [45]	Uc	Yes	Uc	Uc	Yes	Uc	Yes	Uc	Yes	Uc	4	High
Tan et al., 2021 [46]	Uc	Yes	Uc	Uc	Uc	Uc	Uc	Uc	Yes	Uc	2	High
Fu et al., 2021 [47]	Uc	Yes	Uc	Uc	Uc	Uc	Uc	Uc	Yes	Uc	2	High
Kim et al., 2021 [48]	Uc	Yes	Uc	Uc	Uc	Uc	Uc	Uc	Yes	Uc	2	High
Bakhtiar et al., 2022 [49]	Uc	Yes	Yes	Uc	Uc	Uc	Yes	Uc	Yes	Uc	4	High
Zheng et al., 2023 [50]	Uc	Yes	Uc	Uc	Uc	Uc	Uc	Uc	Yes	Uc	2	High
Shi et al., 2023 [51]	Uc	Yes	Uc	Uc	Uc	Uc	Uc	Uc	Yes	Uc	2	High
Bakhtiar et al. [52], 2023	Uc	Yes	Uc	Uc	Yes	Uc	Yes	Uc	Yes	Uc	4	High
Yuan et al. [53], 2023	Uc	Yes	Uc	Uc	Uc	Uc	Uc	Uc	Yes	Uc	2	High

### Synthesis of results

Qualitative analysis of the studies was performed; however, meta-analysis was not feasible due to wide variations amongst studies in methods for assessment, decellularization protocols, animal models used, and nature and source of decellularized ECM.

## Discussion

The field of regenerative endodontics has recently been challenged by a propensity of evidence that demonstrates the difficulty to regenerate true dentin-pulp tissue using clinically relevant scenarios. Currently, it is not clear that any of the conventional protocols of REPs are likely to recapitulate, histologically, the morphological and physiological characteristics of native endodontic tissues [16, 58].

The role of the scaffold has recently changed from being a passive carrier to being a bioactive material with tailored properties for guided regeneration of specific tissues. For an engineered tissue, an ideal scaffold material should mimic the physiological and physical nature of the extracellular matrix of the native target tissue [59]. The dentin-pulp organ is a unique entity with a complex microenvironment.

Normal pulp extracellular matrix (ECM) is a non-mineralized tissue, however, pulp cells express a cocktail of growth factors and cytokines with certain ratios that can regulate mineralization in response to external stimuli [60]. Pulp ECM is predominantly composed of structural proteins in the form of type I and type III collagen (Col-I and Col-III). Other specialized proteins (primarily fibronectin and laminin) and glycosaminoglycans (such as chondroitin sulphate and hyaluronic acid), are also found [61]. The dental pulp serves as a source of nutrition and sensation to dentin. Moreover, it has its own defensive and reparative functions [4].

To date, natural and synthetic biomaterials that can mimic dentin-pulp ECM to reproduce all its complex characteristics are still not available [62]. Therefore it was suggested that decellularized extracellular matrix (ECM)-derived scaffolds could offer a natural biomimetic alternative to conventional scaffolds [23]. Owing to its rich content of tissue-specific growth factors and chemical cues, ECM not only supports cell functions but also dictates cells’ commitment and guides their differentiation lineage [24]. Although many ECM components such as hyaluronic acid [63] and collagen [64] have been tested clinically, whole-tissue decellularized ECM has not been yet accepted for clinical use in regenerative endodontics [23]. It is thus imperative for clinicians and investigators to analyse, via a systematic approach, the variables observed in the preclinical protocols for dECM scaffolding techniques and their effect on the outcomes of regenerative endodontics.

Therefore, the present systematic review was conducted with the aim of studying the role of decellularized ECM-derived scaffolds in dentin-pulp regeneration. However, due to the high risk of bias and average quality of the evidence of the included studies, a meta-analysis was not feasible.

### Histological outcome of using dECM-derived scaffolds for regenerative endodontic applications

The unpredictable regeneration of tissues following conventional regenerative endodontic procedures remains a major concern [65]. Several histological studies using blood-derived scaffolds have reported evidence of ectopic tissue regeneration [16, 65, 66]. Bone-like, cementum-like tissues and absence of odontoblast-like layer are often observed within regenerated tissues [67, 68]. Regenerated tissues appear to be mineralized counterparts of a mixture of tissues such as dentin, bone, cementum and periodontal tissues which have been collectively termed dentin associated mineralized tissues (DAMT) owing to their deposition and close association to the original inner canal wall dentin [69].

Results of this review indicate that decellularized ECM-derived scaffolds are novel biomimetic materials that can lead to enhanced angiogenesis and regeneration of dentin-pulp-like tissues compared to non-ECM-derived scaffolds [23, 38–51]. Notably, the included studies that employed decellularized ECM-derived scaffolds, used other naturally-derived scaffolds as controls rather than synthetic scaffolds [23, 38–51]. It was noted that thirteen of reviewed studies used IHC analysis to detect the expression levels of odontogenic/angiogenic markers. This method of evaluation is valuable for distinguishing the nature and quality of newly-formed tissues. The regenerated tissues were characterized by high expression of DSP and DPP, increased collagen deposition and neovascularization as reported by *Ravidnran *et al. [38] and *Huang *et al. [42] who both used cell-generated dECM scaffolds. These results were also in accordance with the findings of *Chen *et al. [39]*, Zhang *et al. [40]*, Hu *et al. [41]*, Alqahtani *et al. [23] and *Zheng *et al. [50] who all used porcine dental pulp ECM-derived scaffolds. On the contrary, bone/cementum-like hard tissue formation was reported in the study by *Fu *et al. [47] in the acellular ECM group compared to high DSPP and DMP-1 expression in the laminin-coated ECM group. Formation of bone-like tissue was also observed by *Bakhtiar *et al. [43] when crosslinked bovine pulp dECM scaffold was used. This also coincides with the findings of *Kim *et al. [48] who reported hard tissue formation not resembling dentin when human dental pulp dECM was the scaffold, although they reported expression of some odontogenic markers such as DSPP and DMP-1 in the newly formed tissue. Enhanced angiogenesis and detection of pulp-like tissue were reported by *Bakhtiar *et al. [44, 49, 52] when either bovine pulp dECM or amniotic membrane dECM were used as scaffolds.

Generally, in the reviewed studies, it was evident that cell-seeded dECM-derived scaffolds triggered more organized tissue regeneration and robust neovascularization compared to cell-free scaffolds [51] except for one study [52]. However, the fact that those studies have used ectopic or semi-orthotopic models, cannot be overlooked. As the placement of the scaffold out of its original microenvironment may have had a significant effect on host cell recruitment and their behaviour [70]. Indeed, in the study by *Alqahtani *et al. [23], evidence of dentin-pulp regeneration was reported when the cell-homing approach was used in an orthotopic model which indicates the importance of providing the appropriate “niche”.

### Potential contributing factors to histological outcomes

#### Influence of decellularization, lyophilization and terminal sterilization protocols

When dECM-derived scaffold is prepared, it must be mechanically separated from unwanted tissue structures, decellularized, often dehydrated or lyophilized then terminally sterilized [24, 71]. Each of these processing steps can alter the integrity and composition of the matrix [24].

In this review, studies that used treated dentin matrix (TDM) as the main scaffold were not included as it is not considered a decellularized tissue but rather a demineralized tissue [72]. Decellularization can be described as a procedure that aims to remove cellular contents of a tissue, leaving the extracellular matrix free of antigens as well as preserving its original three-dimensional biostructure [24].

A prominent finding to be highlighted in the included records, is the heterogeneity of decellularization and sterilization protocols. Remnants of chemicals and/or enzymes used during decellularization may result in residual cytotoxicity to host cells [24]. It was reported by *Bakhtiar *et al. [44] that SDS-free protocol was more biocompatible compared to SDS-containing protocols. Moreover, higher retention of GAGs and collagen contents was observed when SDS was not used [44]. Other factors to be considered, are lyophilization of the scaffolds and terminal sterilization using ethylene oxide (EtO); these steps were found to affect tissue architecture and decrease growth factor contents of dECM [23]. It can be suggested to use other methods of sterilization such as immersion in penicillin/streptomycin or peracetic acid for better preservation of native biological and structural integrity of dECM [73].

#### Influence of the animal model on regenerative outcomes

Another factor to be considered is the animal host used in each study. The natural response to tissue injury i.e., wound healing cascades, involves a complex sequence of events that includes vascular, cellular and humoral components; the outcome of which is either tissue necrosis and scarring or reconstruction of the tissue with return of function [74]. Decellularized ECM-derived scaffolds can modulate the wound healing response toward constructive remodelling rather than tissue destruction and scar formation [24, 71].

The use of small animal models in most of the reviewed studies might have influenced the regenerative outcomes, as the host response could differ from one species to another. Small animals are often used due to their ease of handling and economical value. Nevertheless, larger animals will eventually be needed to test conditions that highly mimic those in humans, especially regarding working inside the root canal space [75]. Large animals that have dental anatomy and tooth size comparable to humans allow for clinical simulation and evaluation of orthotopic pulp regeneration [75]. Additionally, large animal models can allow the simulation of either short or long-standing infections within the root canal space eventually leading to periapical disease [75]. Such models are critical to be employed since it has been shown that the structure of dentin previously exposed to bacterial biofilms is altered which may negatively affect the migration, attachment, proliferation and differentiation of recruited stem and progenitor cells. Furthermore, infected dentin may sequester variable levels of growth factors as compared to natural healthy dentin [76]. This is in addition to the fact that it has been shown that the larger the periapical lesion and the longer the infection, the higher the virulence of microorganisms thereby creating a more challenging environment for tissue regeneration [77]. The more severe the infection is, the more is the residual inflammatory response and hence this has been shown to have detrimental effects on regenerated tissues following REPs in animal models [78]. Such challenging conditions require the use of more effective antimicrobial strategies which in turn may further negatively affect the regenerative process [77]. The issue of selecting an appropriate model for REPs is indeed a major issue for translational research especially in the tissue engineering and regenerative medicine field.

In the current review, only one study simulated the clinical protocol for REPs [23]. It is worth mentioning that in this study, the root canals were not infected and there were no periapical lesions present prior to treatment which does not truly replicate the clinical scenario [23]. In fact, none of the included studies have used an infected model in their methodologies. Future studies should focus on not only optimizing and assessing the regenerative potential of the scaffold, but also work towards using decellularized ECM-derived scaffolds in animal models that can truly represent translational research as well as in well-executed randomized clinical trials. Moreover, future animal and clinical studies should evaluate the efficacy of dECM-derived scaffolds in the presence of microbial challenge.

#### Influence of scaffold source on regenerative outcome

The alignment and organization of collagen fibres and the concentration of functional proteins are dependent on the native function of the source tissue from which the ECM is derived [24, 71]. Consequently, if dentin/pulp regeneration is the case, the use of dECM of dental origin would be a more suitable choice.

Decellularized extracellular matrix derived from various human or animal tissues has been considered as a possible scaffolding medium for tissue regeneration in present studies.

Most of the reviewed studies used decellularized dental pulp-derived ECM rather than other tissues of dental and non-dental origin. However, none of the studies compared between the tissue sources (dental and non-dental) regarding their biocompatibility and their biological influence on regenerated tissues. Moreover, none of them evaluated the difference between human and xenogeneic sources of ECM scaffolds regarding their in vitro characterization and in vivo regenerative potential. Using an autologous scaffold from discarded third molars or teeth extracted for orthodontic reasons would avoid the problems of antigenicity. However, it will not be feasible in most clinical situations, hence using a xenogeneic form of ECM may be more clinically applicable [71]. Indeed, one of the main concerns of using such scaffolds, could be their possible antigenicity. However, several studies reported that following careful decellularization protocols, minimal immunological response was reported [43, 44, 47, 50, 52]. In studies assessing immunogenicity of dECM scaffolds, no systemic toxicity on major organs and no immune rejection from host tissues were detected, regardless of the source of dECM [43, 44, 47, 50, 52]. This could also be attributed to the immunomodulatory effect of dECM shifing the polarisation of the macrophage population from M1 pro-inflammatory to M2 anti-inflammatory phenotypes resulting in constructive remodelling [71, 79].

#### Influence of scaffold form and method of delivery

Incorporating other materials with decellularized ECM to make composite scaffolds appears to have more predictable results. In the study by *Fu *et al. [47], laminin-modified dECM resulted in regeneration of dentin-like tissue compared to cementum/bone-like tissue in laminin-free dECM group. likewise, dECM incorporating gelatine methacrylate (GelMA) microspheres, resulted in dentin-pulp-like tissue regeneration [50]. However, these favourable outcomes may not be only caused by the direct effect of the added materials, but also due to the modified “physical properties” of the composite scaffold [80]. It has been proven that physical characteristics of the scaffold such as the degree of stiffness and modulus of elasticity can have indirect influence on the commitment of recruited cells and nature of regenerated tissues [80–82]. Regeneration of organized pulp-like tissue was also reported by *Bakhtiar *et al. [52] following the use of cross-linked hydrogel scaffolds. It is worth mentioning that only three studies used the scaffolds as injectable hydrogel form [50, 52, 53]. Future studies should focus on optimizing the form of delivery of the scaffold, i.e., using hydrogels or biphasic scaffolds that might be more logical in regenerative endodontic applications.

Loading dECM with growth factors could also potentiate the action of dECM scaffolds. Addition of BMP4 in the study by *Tan *et al. [46] resulted in organized pulp-like tissue formation. However, this might not be as important in the actual clinical protocol for regenerative endodontic procedures (REPs) when EDTA or other chelating agents are used to release the sequestered growth factors from dentin [83, 84].

### Limitations and future perspectives

In the reviewed studies, there were significant limitations that may have affected outcome assessment. None of the studies compared between dECM-derived scaffolds and blood derived-scaffolds currently used in REPs. There was methodological heterogeneity in the histological assessment and interpretation of results. Additionally, the duration of studies ranged from 2 to 12 weeks in most of the included studies which may not be sufficient time to assess scaffold remodelling and long-term efficacy of the scaffold on dentin-pulp regeneration. Although all included studies had both in vitro and in vivo phases, we only extracted data that was relevant to the in vivo experiments and histological outcomes. The quality and risk of bias assessments indicated that most of reviewed records are preliminary studies lacking sufficient sample size, randomization, blinding and proper statistical analyses. Other limitations of this systematic review include the small number of available studies.

Future studies should work towards optimizing decellularization and sterilization protocols with maximum preservation of ECM architecture and innate growth factors content. Moreover, evaluating the effect of adding natural or synthetic modifiers to the dECM-derived scaffolds should be also addressed, with the purpose of improving specific mechanical characteristics, such as stiffness, viscoelasticity and biodegradation. Additionally, preclinical and animal models should mimic the clinical protocol of REPs with sufficient long-term follow up. The preparation of freeze-dried hydrogel form that can be stored as an off-the-shelf product could also aid in the clinical translation of using dECM-derived scaffolds in REPs. Until this “optimal” scaffold is available, it is important not to lose sight of clinically-relevant outcomes, namely; patient-centered and clinician-centered, as these continue to be measurable and reproducible.

## Conclusions

Decellularized ECM-derived scaffolds could offer a promising biomimetic alternative to current scaffolds used for regenerative endodontic procedures. These cell-free scaffolds may provide comparable histological outcomes to their cell-seeded counterparts thereby offering a potential off-the-shelf scaffold for dentin-pulp regeneration. However, due to the heterogeneity of decellularization methods, animal models, scaffold source, form and delivery, as well as the high risk of bias and average quality of the studies included in this review, the overall effectiveness of decellularized ECM-derived scaffolds still remains uncertain. Therefore, more standardized preclinical research is needed as well as well-constructed clinical trials to prove the efficacy of these scaffolds for clinical translation of organized and functional dentin-pulp regeneration.

### Supplementary Information

**Supplementary Material 1.**

**Supplementary Material 2.**

**Supplementary Material 3.**

**Supplementary Material 4.**

**Supplementary Material 5.**

**Supplementary Material 6.**

## Data Availability

Data is provided within the manuscript or supplementary information files.
