# Supplementary material for: Influence of extracellular matrix scaffolds on histological outcomes of regenerative endodontics in experimental animal models: a systematic review
Source: BMC Oral Health. 2024 Apr 30;24:511. doi: 10.1186/s12903-024-04266-x (PMC11061952; doi:10.1186/s12903-024-04266-x)
Supplement: Supplementary file 1 — Supplementary Material 1. [file 12903_2024_4266_MOESM1_ESM.docx]

**Scopus**

(SUBJAREA ( medi OR nurs OR vete OR dent OR heal OR mult) TITLE-ABS-KEY ( regenerative OR regeneration OR revasculariz* OR revitaliz* AND endodontics OR pulp)) AND ( SUBJAREA ( medi OR nurs OR vete OR dent OR heal OR mult) TITLE-ABS-KEY ( decellularized OR decellulariz* OR extracellular OR matrix OR scaffold OR scaffold*)) AND ( TITLE-ABS-KEY ( histological AND outomes OR histo*))

**Google Scholar**

(((Regenerative Endodontics) OR (Dental Pulp) OR (Revascularization))) AND ((Decellularized Extracellular Matrix) OR scaffold) AND Histo*

**PubMed**

((("Dental Pulp"[MeSH Terms] OR ("Dental Pulp"[MeSH Terms] OR ("dental"[All Fields] AND "pulp"[All Fields]) OR "Dental Pulp"[All Fields])) AND ("revascularisation"[All Fields] OR "revascularisations"[All Fields] OR "revascularise"[All Fields] OR "revascularised"[All Fields] OR "revascularising"[All Fields] OR "revascularization"[All Fields] OR "revascularizations"[All Fields] OR "revascularize"[All Fields] OR "revascularized"[All Fields] OR "revascularizes"[All Fields] OR "revascularizing"[All Fields] OR ("revitalization"[All Fields] OR "revitalize"[All Fields] OR "revitalized"[All Fields] OR "revitalizes"[All Fields] OR "revitalizing"[All Fields]))) OR "pulp revascularization"[All Fields] OR "pulp revitalization"[All Fields] OR ("regenerative"[All Fields] AND ("Endodontics"[MeSH Terms] OR ("endodontal"[All Fields] OR "endodontic"[All Fields] OR "endodontical"[All Fields] OR "endodontically"[All Fields] OR "Endodontics"[MeSH Terms] OR "Endodontics"[All Fields]) OR ("endodontal"[All Fields] OR "endodontic"[All Fields] OR "endodontical"[All Fields] OR "endodontically"[All Fields] OR "Endodontics"[MeSH Terms] OR "Endodontics"[All Fields])))) AND ("Decellularized Extracellular Matrix"[MeSH Terms] OR "Extracellular Matrix"[MeSH Terms] OR "decellulariz*"[All Fields] OR ("scaffold"[All Fields] OR "scaffold s"[All Fields] OR "scaffolded"[All Fields] OR "scaffolder"[All Fields] OR "scaffolders"[All Fields] OR "scaffolding"[All Fields] OR "scaffoldings"[All Fields] OR "scaffolds"[All Fields])) AND ("Histological Techniques"[MeSH Terms] OR "histo*"[All Fields])

**Web of Science**

(((((ALL=(Dental pulp AND (revascularization OR revitalization) )) OR ALL=( “pulp revascularization” OR “pulp revitalization”)) OR ALL=((regenerative AND (“Endodontics” [Mesh] OR endodontics OR endodontic)))) AND (((ALL=(decellulariz*)) OR ALL=(extracellular matrix)) OR ALL=(scaffold))) AND ((ALL=(histo*)) OR ALL=("histological techniques"[mesh])))
