# Supplementary material for: Influence of extracellular matrix scaffolds on histological outcomes of regenerative endodontics in experimental animal models: a systematic review
Source: BMC Oral Health. 2024 Apr 30;24:511. doi: 10.1186/s12903-024-04266-x (PMC11061952; doi:10.1186/s12903-024-04266-x)
Supplement: Supplementary file 2 — Supplementary Material 2. [file 12903_2024_4266_MOESM2_ESM.docx]

List of excluded studies and reasons for exclusion:

| **Author/year** | **Title** | **Reason for exclusion** |
| --- | --- | --- |
| **El-Backly et al^(1)^/ 2008** | Regeneration of dentine/pulp‐like tissue using a dental pulp stem cell/poly (lactic‐co‐glycolic) acid scaffold construct in New Zealand white rabbits | Scaffolds are not decellularized ECM |
| **Yamauchi N et al ^(2)^ /2011** | Immunohistological characterization of newly formed tissues after regenerative procedure in immature dog teeth | Scaffolds are not decellularized ECM |
| **Traphagen SB et al^(3)^/2012** | Characterization of natural decellularized and reseeded porcine tooth bud matrices | In vitro study |
| **Rosa et al^(4)^/ 2013** | Dental pulp tissue engineering in full-length human root canals | Scaffolds are not decellularized ECM |
| **Tawfik et al^(5)^/ 2013** | Regenerative potential following revascularization of immature permanent teeth with necrotic pulps | Scaffolds are not decellularized ECM |
| **Khademi et al^(6)^/ 2014** | Outcomes of revascularization treatment in immature dog's teeth | Scaffolds are not decellularized ECM |
| **Torabinejad et al ^(7)^/2014** | Histologic examinations of teeth treated with 2 scaffolds: a pilot animal investigation | Scaffolds are not decellularized ECM |
| **Torabinejad et al^(8)^/ 2015** | Histologic examination of teeth with necrotic pulps and periapical lesions treated with 2 scaffolds: an animal investigation | Scaffolds are not decellularized ECM |
| **Dissanayaka et al^(9)^ /2015** | The interplay of dental pulp stem cells and endothelial cells in an injectable peptide hydrogel on angiogenesis and pulp regeneration in vivo | Scaffolds are not decellularized ECM |
| **Londero et al^(10)^/ 2015** | Histologic analysis of the influence of a gelatin-based scaffold in the repair of immature dog teeth subjected to regenerative endodontic treatment | Scaffolds are not decellularized ECM |
| **Iwamoto et al^(11)^/ 2016** | Tooth tissue engineering: tooth decellularization for natural scaffold | In vitro study |
|  |  |  |
| **Ahuja et al ^(12)^/2017** | Development of a Demineralized Dentin Matrix Hydrogel for Dental Pulp Regeneration | Scaffolds are not decellularized ECM |
| **Matoug‐Elwerfelli et al ^(13)^ /2017** | Decellularisation of the Dental Pulp for Use as a Scaffold in Regenerative Endodontics | In vitro study |
| **Fahmy et al^(14)^/ 2017** | Investigation of the regenerative potential of necrotic mature teeth following different revascularisation protocols | Scaffolds are not decellularized ECM |
| **Song J.S. et al^(15)^ /2017** | Decellularized Human Dental Pulp as a Scaffold for Regenerative Endodontics | In vitro study |
| **Matoug‐Elwerfelli et al ^(16)^/2018** | A biocompatible decellularized pulp scaffold for regenerative endodontics | In vitro study |
| **El Ashiry et al^(17)^/ 2018** | Tissue engineering of necrotic dental pulp of immature teeth with apical periodontitis in dogs: radiographic and histological evaluation | Scaffolds are not decellularized ECM |
| **Wang F. et al ^(18)^/2019** | Human Freeze-dried Dentin Matrix as a Biologically Active Scaffold for Tooth Tissue Engineering | Scaffolds are not decellularized ECM |
| **El Kalla et al^(19)^/ 2019** | Histological evaluation of platelet-rich fibrin for revascularization of immature permanent teeth in dogs | Scaffolds are not decellularized ECM |
|  |  |  |
| **Chang C. et al^(20)^ /2020** | Regeneration of Tooth with Allogenous, Autoclaved Treated Dentin Matrix with Dental Pulpal Stem Cells: An In Vivo Study | Scaffolds are not decellularized ECM |
| **Ling et al^(21)^/ 2020** | Regeneration of dental pulp tissue by autologous grafting stem cells derived from inflammatory dental pulp tissue in immature premolars in a beagle dog | Scaffolds are not decellularized ECM |
| **Matoug-Elwerfelli et al ^(22)^/2020** | Ex-vivo recellularisation and stem cell differentiation of a decellularised rat dental pulp matrix | Ex-vivo study |
| **El Halaby et al^(23)^/ 2020** | Evaluation of the regenerative potential of dentin conditioning and naturally derived scaffold for necrotic immature permanent teeth in a dog model | Scaffolds are not decellularized ECM |
| **Siddiqui et al ^(24)^/2021** | Angiogenic hydrogels for dental pulp revascularization | Scaffolds are not decellularized ECM |
| **Alenazy et al^(25)^/ 2021** | Histologic, radiographic, and micro-computed tomography evaluation of experimentally enlarged root apices in dog teeth with apical periodontitis after regenerative treatment | Scaffolds are not decellularized ECM |
| **Sequeira et al^(26)^/ 2021** | Regeneration of pulp-dentin complex using human stem cells of the apical papilla: In vivo interaction with two bioactive materials | Scaffolds are not decellularized ECM |
| **Diomede F. et al ^(27)^/2022** | Decellularized Dental Pulp, Extracellular Vesicles, and 5‐Azacytidine: A New Tool for Endodontic Regeneration | In vitro study |
|  |  |  |
| **AlHowaish et al^(28)^/ 2022** | Histological evaluation of restylane lyft used as a scaffold for dental pulp regeneration in non-infected immature teeth in dogs | Scaffolds are not decellularized ECM |
| **Ribeiro et al^(29)^/ 2024** | Histologic and proteomic profile of two methods to decellularize human dental pulp tissue. Archives of Oral Biology | In vitro study |
| **Han et al^(30)^/ 2024** | Injectable Tissue-Specific Hydrogel System for Pulp–Dentin Regeneration | Scaffolds are not decellularized ECM |

**References:**

1. El‐Backly RM, Massoud AG, El‐Badry AM, Sherif RA, Marei MK. Regeneration of dentine/pulp‐like tissue using a dental pulp stem cell/poly (lactic‐co‐glycolic) acid scaffold construct in New Zealand white rabbits. Australian Endodontic Journal. 2008;34(2):52-67.

2. Yamauchi N, Nagaoka H, Yamauchi S, Teixeira FB, Miguez P, Yamauchi M. Immunohistological characterization of newly formed tissues after regenerative procedure in immature dog teeth. Journal of endodontics. 2011;37(12):1636-41.

3. Traphagen SB, Fourligas N, Xylas JF, Sengupta S, Kaplan DL, Georgakoudi I, et al. Characterization of natural, decellularized and reseeded porcine tooth bud matrices. Biomaterials. 2012;33(21):5287-96.

4. Rosa V, Zhang Z, Grande RHM, Nör J. Dental pulp tissue engineering in full-length human root canals. Journal of dental research. 2013;92(11):970-5.

5. Tawfik H, Abu‐Seida A, Hashem A, Nagy M. Regenerative potential following revascularization of immature permanent teeth with necrotic pulps. International endodontic journal. 2013;46(10):910-22.

6. Khademi AA, Dianat O, Mahjour F, Razavi SM, Younessian F. Outcomes of revascularization treatment in immature dog's teeth. Dental Traumatology. 2014;30(5):374-9.

7. Torabinejad M, Faras H, Corr R, Wright KR, Shabahang S. Histologic examinations of teeth treated with 2 scaffolds: a pilot animal investigation. Journal of endodontics. 2014;40(4):515-20.

8. Torabinejad M, Milan M, Shabahang S, Wright KR, Faras H. Histologic examination of teeth with necrotic pulps and periapical lesions treated with 2 scaffolds: an animal investigation. Journal of endodontics. 2015;41(6):846-52.

9. Dissanayaka WL, Hargreaves KM, Jin L, Samaranayake LP, Zhang C. The interplay of dental pulp stem cells and endothelial cells in an injectable peptide hydrogel on angiogenesis and pulp regeneration in vivo. Tissue Engineering Part A. 2015;21(3-4):550-63.

10. Londero CdLD, Pagliarin CML, Felippe MCS, Felippe WT, Danesi CC, Barletta FB. Histologic analysis of the influence of a gelatin-based scaffold in the repair of immature dog teeth subjected to regenerative endodontic treatment. Journal of Endodontics. 2015;41(10):1619-25.

11. Iwamoto LAdS, Duailibi MT, Juliano Y, Duailibi MS, Tanaka FAO, Duailibi SE. Tooth tissue engineering: tooth decellularization for natural scaffold. Future Science OA. 2016;2(2):1-9.

12. Ahuja N. Development of a Demineralized Dentin Matrix Hydrogel for Dental Pulp Regeneration 2017.

13. Matoug-Elwerfelli M. Decellularisation of the Dental Pulp for Use as a Scaffold in Regenerative Endodontics: University of Leeds; 2017.

14. Fahmy SH, Hassanien EES, Nagy MM, El Batouty KM, Mekhemar M, Fawzy El Sayed K, et al. Investigation of the regenerative potential of necrotic mature teeth following different revascularisation protocols. Australian Endodontic Journal. 2017;43(2):73-82.

15. Song J, Takimoto K, Jeon M, Vadakekalam J, Ruparel N, Diogenes A. Decellularized human dental pulp as a scaffold for regenerative endodontics. Journal of dental research. 2017;96(6):640-6.

16. Matoug‐Elwerfelli M, Duggal M, Nazzal H, Esteves F, Raïf E. A biocompatible decellularized pulp scaffold for regenerative endodontics. International Endodontic Journal. 2018;51(6):663-73.

17. El Ashiry EA, Alamoudi NM, El Ashiry MK, Bastawy HA, El Derwi DA, Atta HM. Tissue engineering of necrotic dental pulp of immature teeth with apical periodontitis in dogs: radiographic and histological evaluation. Journal of Clinical Pediatric Dentistry. 2018;42(5):373-82.

18. Wang F, Xie C, Ren N, Bai S, Zhao Y. Human freeze-dried dentin matrix as a biologically active scaffold for tooth tissue engineering. Journal of Endodontics. 2019;45(11):1321-31.

19. El Kalla IH, Salama NM, Wahba AH, Sallam NM. Histological evaluation of platelet-rich fibrin for revascularization of immature permanent teeth in dogs. Pediatric Dental Journal. 2019;29(2):72-7.

20. Chang C-C, Lin T-A, Wu S-Y, Lin C-P, Chang H-H. Regeneration of tooth with allogenous, autoclaved treated dentin matrix with dental pulpal stem cells: an in vivo study. Journal of Endodontics. 2020;46(9):1256-64.

21. Ling L, Zhao YM, Wang XT, Wen Q, Ge LH. Regeneration of dental pulp tissue by autologous grafting stem cells derived from inflammatory dental pulp tissue in immature premolars in a beagle dog. Chin J Dent Res. 2020;23(2):143-50.

22. Matoug-Elwerfelli M, Nazzal H, Raif EM, Wilshaw S-P, Esteves F, Duggal M. Ex-vivo recellularisation and stem cell differentiation of a decellularised rat dental pulp matrix. Scientific Reports. 2020;10(1):21553.

23. El Halaby HM, Abu‐Seida AM, Fawzy MI, Farid MH, Bastawy HA. Evaluation of the regenerative potential of dentin conditioning and naturally derived scaffold for necrotic immature permanent teeth in a dog model. International Journal of Experimental Pathology. 2020;101(6):264-76.

24. Siddiqui Z, Sarkar B, Kim K-K, Kadincesme N, Paul R, Kumar A, et al. Angiogenic hydrogels for dental pulp revascularization. Acta biomaterialia. 2021;126:109-18.

25. Alenazy MS, Al-Nazhan S, Mosadomi HA. Histologic, radiographic, and micro-computed tomography evaluation of experimentally enlarged root apices in dog teeth with apical periodontitis after regenerative treatment. Current Therapeutic Research. 2021;94:100620.

26. Sequeira DB, Oliveira AR, Seabra CM, Palma PJ, Ramos C, Figueiredo MH, et al. Regeneration of pulp-dentin complex using human stem cells of the apical papilla: In vivo interaction with two bioactive materials. Clinical Oral Investigations. 2021;25:5317-29.

27. Diomede F, Fonticoli L, Marconi GD, Della Rocca Y, Rajan TS, Trubiani O, et al. Decellularized Dental Pulp, Extracellular Vesicles, and 5-Azacytidine: A New Tool for Endodontic Regeneration. Biomedicines. 2022;10(2):403.

28. AlHowaish NA, AlSudani DI, Khounganian R, AlMuraikhi N. Histological evaluation of restylane lyft used as a scaffold for dental pulp regeneration in non-infected immature teeth in dogs. Materials. 2022;15(12):4095.

29. Ribeiro VMdS, Sousa MGdC, Garcia PR, Santos LSd, Duarte ECB, Corrêa JR, et al. Histologic and proteomic profile of two methods to decellularize human dental pulp tissue. Archives of Oral Biology. 2024;157:1-8.

30. Han Y, Xu J, Chopra H, Zhang Z, Dubey N, Dissanayaka W, et al. Injectable Tissue-Specific Hydrogel System for Pulp–Dentin Regeneration. Journal of Dental Research. 2024:00220345241226649.
