# Supplementary material for: Influence of extracellular matrix scaffolds on histological outcomes of regenerative endodontics in experimental animal models: a systematic review
Source: BMC Oral Health. 2024 Apr 30;24:511. doi: 10.1186/s12903-024-04266-x (PMC11061952; doi:10.1186/s12903-024-04266-x)
Supplement: Supplementary file 3 — Supplementary Material 3. [file 12903_2024_4266_MOESM3_ESM.docx]

| Author / Year of publication  Supplementary table 1: General characteristics of the included studies | Type of experiment | In vitro experiments | In vivo experiments | Study findings |
| --- | --- | --- | --- | --- |
| Ravindran et al, 2014 ^(43)^ | - In vitro - In vivo | -IHC (tubulin, FN, BMP-2, TGF𝛽, VEGF, MMP2, MMP9, phospho serine and phosphor tyrosine, DMP1, DSP, DPP, thrombospondin and von Willebrand factor)  RT-qPCR: In vitro differentiation of DPSCs (with or without DPP) and PDLSCs on ECM scaffold | - Subcutaneous implantation of the scaffold | - DPP blocking led to reduced expression of many genes expressed by differentiating DPSCs. - The scaffold stained positive for Growth factors such as BMP2, TGFβ, VEGF and PEDF - Subcutaneous implantation of the ECM scaffolds containing DPSCs showed the formation of dental pulp-like tissue with cells expressing DSP and DPP. |
| Chen et al, 2015 ^(44)^ | - In vitro - In vivo | - SEM analysis  -IF by DAPI  -IHC: for ECM proteins (Col-I, Col-III, FN and LN)  -Cell proliferation and cell infiltration detected by IF “DAPI” and SEM.  -RT-qPCR: odontogenic influence on hDFSCs | - Ectopic subcutaneous implantation (angiogenesis) - Semi-orthotopic implantation in jaw bones (regeneration) | - After decellularization, dECM retained the size, shape and original consistency of dental pulp tissues. - Col-1 and Col-3 showed significantly decline in DPEM, especially in odontoblastic layer. FN didn't appear to decrease, and it was significantly expressed in dECM - Cell morphology changed after 7 days incubation with dECM. scaffold upregulated BSP DMP-1 and DSPP expression, retained VEGF expression and downregulated ALP and RUNX-2. - dECM enhanced revascularization and triggered the formation of pulp-like tissue. |
| Zhang et al, 2017 ^(45)^ | - In vitro - In vivo | -IF analysis (Vimentin, E-cadherin and Factor VIII): to confirm the survival of seeded cells prior to scaffold implantation.  -Cell isolation and culture then seeding of cell into dECM scaffold. | - Semi-orthotopic implantation in extraction socket | - The majority of cells were Vimentin positive - E-cadherin–positive cells were localized to the periphery of the construct. - Factor VIII–positive cells were detected throughout the in vitro cultured constructs - Recell-dTB constructs consisted of ~75% hDPCs, 10% dental epithelial cells, and 19% HUVECs. - BMP2+dTBs group: no calcific tissues formed. - Acellular dTBs group and native TBs group: pulp+dentin but no enamel - -Recell-dTBs groups: enamel+dentin+pulp, cementum, and periodontal ligament. |
| Hu et al, 2017 ^(46)^ | - In vitro - In vivo | -SEM analysis  -IF staining for (Col-IV, laminin, fibronectin, integrin 𝛽1, and vimentin)  -Cell isolation, culture and seeding into dECM scaffold. | - Subcutaneous semi-orthotopic implantation in tooth slices | - ECM retained its protein content of Col-IV, laminin, fibronectin and integrin 𝛽1. - Vimentin expression was decreased and retained only in the odontoblast layer. - Newly formed pulp-like and mineralized tissues were detected in vivo. |
| Alqahtani et al, 2018 ^(24)^ | - In vitro - In vivo | -SEM analysis  -IF staining for (Col-I, DSP, DMP-1 and vWF).  -Growth factors quantification by ELISA (VEGF, bFGF and TGF-β1)  -Proliferation and migration of DPSCs on digested dECM.  . | - Orthotopic placement of scaffolds in necrotic teeth | - SEM of the native tissue and DP-ECM shows changes in the tissue architecture after decellularization. - ECM components were still detectable with IF after decellularization. - Seven days after treatment, DPSCs maintained a proliferation rate similar to the control groups. - Cell migration was comparable to control groups. - Reduced VEGF and TGF-β1 and non-detectable levels of bFGF after lyophilization and EtO sterilization. - Intracanal pulp-like tissue was observed in vivo. |
| Huang et al, 2018 ^(47)^ | - In vitro - In vivo | -IHC: (FN, DMP-1, DPP, DSP, TGF-β1, BMP-2, vWF, VEGF and bFGF) compared to pulp ECM (control).  -Proliferation of hDPSCs and hBMSCs on pulp ECM and dual ECM  -RT-qPCR for odontogenic and angiogenic markers | - Subcutaneous semi-orthotopic implantation in tooth slices | - Cell proliferation was maintained using both scaffolds. - Both scaffolds contained proteins required for odontogenic differentiation such as Fibronectin, dentin matrix protein 1 (DMP1), dentin phosphophoryn (DPP), dentin sialoprotein (DSP), and bone morphogenic protein 2 (BMP2). - Angiogenic factors were higher in the dual-ECM scaffold. - Both scaffolds promoted robust odontogenic differentiation and vascularization when compared to respective controls. |
| Bakhtiar et al, 2020 ^(48)^ | - In vitro - In vivo | -SEM analysis  -Porosity  -Absorption  -Degradation test  -Cell attachment and morphology (SEM)  -Cytotoxicity and proliferation assay  -Differentiation (RT-qPCR: DSPP, DMP-1 and Col-I) | - Immunogenicity | - The pore size and structural integrity of all the scaffolds were demonstrated. - Concentration of 1.50 mg/ml scaffold demonstrated the highest absorption - All samples demonstrated more than 97% porosity - Concentration of 3.00 mg/ml scaffold degradation was significantly higher than other two concentrations. - Degradation rate of crosslinked scaffold was slower but evoked less inflammation than non-crosslinked scaffolds. - No cytotoxic effects were detected. - Cell proliferation was greater than controls. - Cultured cells were successfully attached to the scaffold surfaces - Higher concentration (3.00 mg/ml) crosslinked scaffold enhanced cell proliferation, promoted their attachment and increased DMP-1 and Col-I expression. - implanted scaffolds revealed low immunological response, and enhanced angiogenesis in cross-linked samples compared tonon-crosslinked samples. |
| Bakhtiar et al, 2020 ^(48)^ | - In vitro - In vivo | - Optimization od decellularization protocol:  Protocol 1: 1-hour EDTA/Trypsin, 24 hours SDS and 1-hour TritonX-100  Protocol 2: 1-hour EDTA/Trypsin, 24 hours SDS and no TritonX-100  -Protocol 3: no EDTA/Trypsin, 48 hours SDS and 1-hour TritonX-100  -Protocol 4: 1-hour EDTA/Trypsin, 48 hours SDS and no TritonX-100  -Protocol 5: 1-hour EDTA/Trypsin, 48 hours SDS and 1-hour TritonX-100  -Protocol 6: 24-hour EDTA/Trypsin, no SDS and 1-hour TritonX-100  - Protocol 7: 12-hour EDTA/Trypsin, no SDS and 1-hour TritonX-100  -IF and DAPI only for Protocol 7 (12E-0S-1T) protocol samples were chosen based on DNA content and histological results. | - Immunogenicity | - All groups showed significantly lower DNA content compared to native pulp tissue - Protocol 7 resulted in highest GAGs content and highest retention of Col-I - The implanted materials (protocol 7) gradually degraded over 14 days and were replaced by new tissues - Highly vascularized connective tissues and fibrous encapsulation were evident |
| Alghutaimel et al, 2021 ^(50)^ | - In vitro - In vivo | -SEM analysis  -IHC (VEGFA and FGF-2)  -Fluorescent staining and confocal scanning laser microscopy for the recellularized ECM.  -Isolation and culture  -Recellularization  -RT-qPCR for the recellularized ECM | - Subcutaneous semi-orthotopic implantation in root segments | - dECM retained its native architecture. - Positive immunoreactivity for VEGFA and FGF-2 in both scaffold and control groups - RT-qPCR revealed enhanced expression of markers involved in angiogenesis - Evidence of vascularization in both seeded or unseeded dECM. - Seeding dECM with hDPSCs has led to more organized cell repopulation and enhanced vascularization capacity. |
| Tan et al, 2021 ^(51)^ | - In vitro - In vivo | -SEM analysis  -Cell proliferation  -Cell differentiation (ALP and alizarin red)  -RT-qPCR for odontogenic/osteogenic and angiogenic genes | - Subcutaneous ectopic implantation | - dECM structure retained its porous fibrous collagen network. - Increased cell proliferation compared to controls. - dECM resulted in increased angiogenic and odontogenic/osteogenic markers compared to cells in normal culture conditions. - The expression of osteogenic and angioblastic genes was increased, and pulp-like tissue formed in vivo. |
| Fu et al, 2021 ^(52)^ | - In vitro - In vivo | -SEM analysis before and after cell seeding  -IF analysis for detection of laminin before and after cell seeding  -Cell adhesion  -RT-qPCR for odontogenic/osteogenic differentiation | - Semi-orthotopic implantation in jaw-bones | - SEM demonstrated that the microstructure of dental pulp tissues possessed a dense surface, but the dECM (Figure presented a loose and porous structure - Laminin modified dECM at the concentration of 100 mg/mL promoted cell adhesion - Increased expression of relative quantification to the early odontogenic genes - No obvious immune rejection or severe inflammatory reaction was observed. |
| Kim et al, 2021 ^(53)^ | - In vivo - In vitro | - Recellularization of scaffold without in vitro characterization | - Subcutaneous semi-orthotopic implantation | - Increased expression of genes related to revascularization hard tissue regeneration. - The expression levels of DSPP, DMP1, Nestin, Col I, ALP, VEGF CD31, and CD34 were significantly higher in the recellularized group than in cell-free group. |
| Bakhtiar et al, 2022 ^(54)^ | - In vitro - In vivo | -Pore size (SEM)  -Porosity  -Absorption  -Degradation  -Cytotoxicity  -Proliferation  -Adhesion and morphology  -Migration | - Immunogenicity - Pulp regeneration | - Scaffold's structure was more homogenous after crosslinking, and the pore size decreased. - All concentrations had more than 95% porosity - No significant difference between groups regarding absorption. - Degradation rate was greater in higher concentration group. - Scaffold maintained cell viability, increased proliferation, attachment and migration compared to controls. - 30 mg/ml ECM scaffold revealed mild to moderate inflammatory response. In root segments, both cell-free and cell-loaded 30 mg/ml scaffolds were replaced with newly formed, pulp-like tissue |
| Zheng et al, 2023 ^(55)^ | - In vitro - In vivo | -SEM analysis  -Degradation rate  -IF (Ki67, DSPP and *β*- III tubulin)  -Cytocompatibility (live/dead assay and DAPI)  -RT‑qPCR (odontogenic genes: RUNX2, DSPP and DMP-1 and for angiogenic genes: CD31, VEGF and for neurogenic gene: nestin). | - Subcutaneous semi-orthotopic implantation | - SEM images revealed that the surfaces of dECM exhibited 3D porous structures after freeze-drying - Complete degradation was accomplished on day 4 in the presence of collagenase. - dECM maintained cell viability and increased their proliferation (increased Ki67 marker) - Enhanced neurogenic and odontogenic markers was detected in response to dECM scaffold - No obvious systemic toxicity of either dECM/GelMA on major organs |
| Shi et al,  2023 ^(56)^ | - In vitro - In vivo | -SEM analysis  -IF (Col-I, Col-III and FN)  -Isolation and culture of DPSCs  -Cell adhesion and proliferation on lyophilized DSMG  -Cell differentiation by RT-qPCR for odontogenic markers. | - Subcutaneous semi-orthotopic implantation in tooth slices | - Immunolabeling of COL1, COL3, and fibronectin revealed comparable ECM components in the native submandibular gland and dental pulp - DSMG retained essential ECM components and architecture, such as collagen and fibronectin. - Cell based analysis indicated that decellularization and lyophilization had no destructive effect on bioactivities of the dECM. - The prepared scaffold supported cell adhesion and proliferation and increased their odontogenic differentiation capacity. - DSMG promoted angiogenesis and dentinogenesis in vivo |
| Bakhtiar et al^(59)^, 2023 | - In vitro - In vivo. | - SEM analysis - Degradation - Rheological analysis of the hydrogel - Tooth discoloration   Cell based analysis:   - Viability of DPSCs - Proliferation of DPSCs | - Immunogenicity - Pulp regeneration | - Non crosslinked hydrogels degraded after 14 days while crosslinked did not degrade up to 28 days - All hydrogels were porous - Crosslinking did not alter rheological characteristic of the scaffold - Regarding discoloration, all groups showed insignificant change of color up 2t 28 days except teeth with blood clot. - All scaffold, crosslinked and non crosslinked, maintained cell viability and increased proliferation - Hydrogels were biocompatible and supported angiogenesis without evident immunogenicity. - Highly vascularized pulp-like tissue formation was observed in 30 mg/ml. |
| Yuan et al^(60)^, 2023 | - In vitro - In vivo | - SEM analysis - Rheological analysis of the hydrogel - Swelling and degradation   Cell based analysis:   - Proliferation - Viability - Migration - Differentiation (RT-qPCR) | - Subcutaneous semi-orthotopic implantation in TDM | - dECM retained its architecture - dECM and GelMA hydrogels showed desirable rheological characteristics. - dECM hydrogels demonstrated favorable biocompatibility and chemotactic activity, and facilitated cell survival, proliferation, and migration - dECM Hydrogels showed robust angiogenic potential and promoted hDPSCs odontogenic and neurogenic differentiation - dECM hydrogels promote regeneration of pulp-like tissue in vivo |

**Abbreviations**

**AB:** alcian blue, **ALP**: alkaline phosphatase, **APES**: aligned Poly(D,Llactide-co-glycolide)/gelatin electrospun sheet, **bFGF**: basic fibroblast growth factor, **BMP-2**: bone morphogenetic protein-2, **BMP-4**: bone morphogenetic protein-4, **CD31**: cluster of differentiation-31, **CD34**: cluster of differentiation-34, **CD68**: cluster of differentiation-68, **Col**: collagen, **CP23**: cementum-derived protein 23, **DAPI**: 4',6-diamidino-2-phenylindole, **dECM**: decellularized extracellular matrix, **dp-ECM**: decellularized pulp extracellular matrix, **dPDL-ECM**: decellularized periodontal ligament extracellular matrix, **DPP**: dentin phosphoprotein, **DPSCs**: human dental pulp stem cells**, DSMG**: decellularized submandibular gland, **DSP**: dentin sialoprotein, **DSPP**: dentin sialophospho protein, **DMP-1**: dentin matric protein 1, **dTBs**: decellularized tooth buds, **FN**: fibronectin, **GAGs**: glycosaminoglycans, **GelMA**: gelatine methacrylate, **HAM**: human amniotic membrane, **hBMSCs**: human bone marrow stem cells, , **hDPSCs**: human dental pulp stem cells, **H&E**: hematoxylin and eosin, **HUVECs**: human umbilical vein endothelial cells, **HN**: human nuclei, **IF**: immunofluorescence, **IHC**: immunohistochemistry, **LN**: laminin, **MMP**: matrix metalloproteinase, **MT**: Masson’s trichrome, **nTB**: native tooth bud, **OC**: osteocalcin, **PBS**: phosphate buffered saline, **Recell-dTB**: recellularized dental tooth bud, **RT**-**qPCR**: reverse transcriptase quantitative polymerase chain reaction, **SDS**: sodium dodecyl sulphate, **SEM**: scanning electron microscope, **SMA**: smooth muscle actin, **TBs**: tooth buds, **TDM**: treated dentin matrix, **TGF𝛽**: transforming growth factor beta, **VEGF**: vascular endothelial growth factor, **vWF**: Von Willebrand factor
