# Supplementary material for: Influence of extracellular matrix scaffolds on histological outcomes of regenerative endodontics in experimental animal models: a systematic review
Source: BMC Oral Health. 2024 Apr 30;24:511. doi: 10.1186/s12903-024-04266-x (PMC11061952; doi:10.1186/s12903-024-04266-x)
Supplement: Supplementary file 6 — Supplementary Material 6. [file 12903_2024_4266_MOESM6_ESM.docx]

**Appendix 1:**

List of abbreviations:

| **Abbreviations** | **Words** |
| --- | --- |
| **AB** | Alcian blue |
| **ALP** | Alkaline phosphatase |
| **APES** | Aligned Poly(D,Llactide-co-glycolide)/gelatin electrospun sheet |
| **bFGF** | Basic fibroblast growth factor |
| **BMP-2** | Bone morphogenetic protein-2 |
| **BMP-4** | Bone morphogenetic protein-4 |
| **CD31** | Cluster of differentiation-31 |
| **CD34** | Cluster of differentiation-34 |
| **CD68** | Cluster of differentiation-68 |
| **Col** | Collagen |
| **CP23** | Cementum-derived protein 23 |
| **DAMT** | Dentin associated mineralized tissues |
| **DAPI** | 4',6-diamidino-2-phenylindole |
| **dECM** | Decellularized extracellular matrix |
| **dp-ECM** | Decellularized pulp extracellular matrix |
| **dPDL-ECM**: | Decellularized periodontal ligament extracellular matrix |
| **DPP** | Dentin phosphoprotein |
| **DSMG** | decellularized submandibular gland |
| **DSP** | Dentin sialoprotein |
| **DSPP** | Dentin sialophospho protein |
| **DMP-1** | Dentin matric protein 1 |
| **dTBs** | Dental tooth buds |
| **EDTA** | Ethylenediamine tetraacetic acid |
| **ECM** | Extracellular matrix |
| **EtO** | Ethylene oxide |
| **FN** | Fibronectin |
| **GAGs** | Glycosaminoglycans |
| **GelMA** | Gelatine methacrylate |
| **HAM** | Human amniotic membrane |
| **hBMSCs** | Human bone marrow stem cells |
| **hDFSCs** | Human dental follicle stem cells |
| **hDPCs** | Human dental pulp cells |
| **hDPSCs** | Human dental pulp stem cells |
| **H&E**: | Hematoxylin and eosin |
| **HUVECs** | Human umbilical vein endothelial cells |
| **HN** | Human nuclei |
| **IF** | Immunofluorescence |
| **IHC** | Immunohistochemistry |
| **Ki67** | Marker of proliferation |
| **LN** | Laminin |
| **MMP** | Matrix metalloproteinase |
| **MSCs** | Mesenchymal stem cells |
| **MT** | Masson’s trichrome |
| **MTT** **assay** | 3-(4,5-dimethylthiazol-2-yl)-2,5-diphenyl-2H-tetrazolium bromide |
| **nTB** | Native tooth bud |
| **OC** | Osteocalcin |
| **OPN** | Osteopontin |
| **PBS** | Phosphate buffered saline, |
| **pDFSCs** | Porcine dental follicle stem cells |
| **QC** | Quality coefficient |
| **Recell-dTB** | Recellularized decellularized tooth bud |
| **RoB** | Risk of bias |
| **RT**-**qPCR** | Reverse transcriptase quantitative polymerase chain reaction |
| **REPs** | Regenerative endodontic procedures |
| **RUNX-2** | Runt-related transcription factor 2 |
| **SDS** | Sodium dodecyl sulphate |
| **TB** | Tooth bud |
| **TDM** | Treated dentin matrix |
| **SEM** | Scanning electron microscope |
| **SMA** | Smooth muscle actin |
| **TDM** | Treated dentin matrix |
| **TGF𝛽** | Transforming growth factor beta |
| **TX-100** | Triton X-100 |
| **VEGF** | Vascular endothelial growth factor |
| **vWF**: | Von Willebrand factor |
